# Polyhydroxylated Steroids from the South China Sea Soft Coral *Sarcophyton* sp. and Their Cytotoxic and Antiviral Activities

**DOI:** 10.3390/md11124788

**Published:** 2013-12-02

**Authors:** Kai-Kai Gong, Xu-Li Tang, Gang Zhang, Can-Ling Cheng, Xing-Wang Zhang, Ping-Lin Li, Guo-Qiang Li

**Affiliations:** 1 Key Laboratory of Marine Drugs, Chinese Ministry of Education, School of Medicine and Pharmacy, Ocean University of China, Yushan Road 5, Qingdao 266003, China; E-Mails: gongkaikai1005@163.com (K.-K.G.); cebianruan@163.com (G.Z.); canling2010@126.com (C.-L.C.); 735888678@qq.com (X.-W.Z.); 2 College of Chemistry and Chemical Engineering, Ocean University of China, Songling Road 238, Qingdao 266100, China; E-Mail: tangxuli@ouc.edu.cn

**Keywords:** soft coral, *Sarcophyton* sp., polyhydroxylated steroids, cytotoxicities, anti-H1N1 activity

## Abstract

Chemical investigation on the soft coral *Sarcophyton* sp. collected from the South China Sea yielded three new polyhydroxylated steroids, compounds (**1**–**3**), together with seven known ones (**4**–**10**). Their structures were established by extensive spectroscopic methods and comparison of their data with those of the related known compounds. All the isolates possessed the 3β,5α,6β-trihydroxylated steroidal nucleus. The cytotoxicities against selected HL-60, HeLa and K562 tumor cell lines and anti-H1N1 (Influenza A virus (IAV)) activities for the isolates were evaluated. Compounds **2**, **3** and **5**–**8** exhibited potent activities against K562 cell lines with IC_50_ values ranging from 6.4 to 10.3 μM. Compounds **1**, **6**–**8** potently inhibited the growth of HL-60 tumor cell lines, and **6** also showed cytotoxicity towards HeLa cell lines. In addition, preliminary structure-activity relationships for the isolates are discussed. The OAc group at C-11 is proposed to be an important pharmacophore for their cytotoxicities in the 3β,5α,6β-triol steroids. Compounds **4** and **9** exhibited significant anti-H1N1 IAV activity with IC_50_ values of 19.6 and 36.7 μg/mL, respectively.

## 1. Introduction

**Figure 1:**
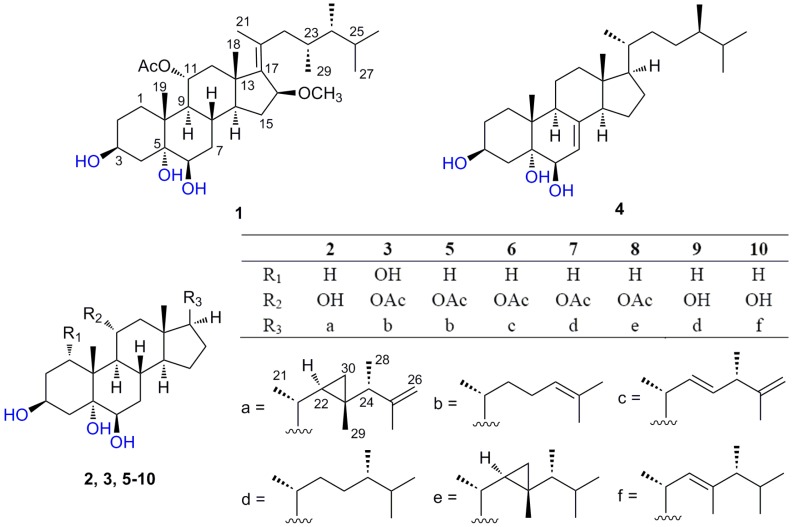
Structures of compounds **1**–**10**.

Polyhydroxylated steroids with a broad spectrum of bioactivities have been found in various phyla of marine organisms, and especially in the subclass Octocorallia, commonly called “soft corals” [1]. The genus *Sarcophyton* was revised to contain 35 valid species, but six additional species have subsequently been added [2]. Previous chemical investigations showed that the *Sarcophyton* is a prolific source of cembrane-type diterpenoids [3], bis-cembranoids [4], and polyhydroxysterols [5]. Recently, a series of structurally diverse polyhydroxysterols with antimicrobial activities were isolated from *Sarcophyton* sp. [6]. In our search for bioactive compounds from marine organisms, we obtained an unidentified species of *Sarcophyton* collected from Weizhou Island of Guangxi Province in the South China Sea. Combined chemistry and bioassay-guided quick isolation of the sterols-rich portion of the MeOH extract of *Sarcophyton* sp., yielded three new polyhydroxysterols, compounds (**1**–**3**), along with seven known analogs (**4**–**10**) (Figure 1). All the isolates possessed the characteristic 3β,5α,6β-trihydroxy moiety, but varied in the side chains and substituted patterns in the nucleus. Structurally, these sterols could be divided into four types belonging to cholesterol, ergosterol, gorgosterol and 23,24-dimethyl cholesterol, of which compound **1** possessed a unique 17(20)-en-23,24-dimethyl side chain, and compound **2** was the firstly discovered gorgosterol with a 25-ene side chain. Their cytotoxicities against three selected tumor cell lines and anti-H1N1 IAV activities were evaluated. Herein, we describe the isolation, structure elucidation of the new polyhydroxysterols (**1**–**3**) and preliminary structure-activity relationships for the isolates (**1**–**10**).

## 2. Results and Discussion

**Figure 2:**
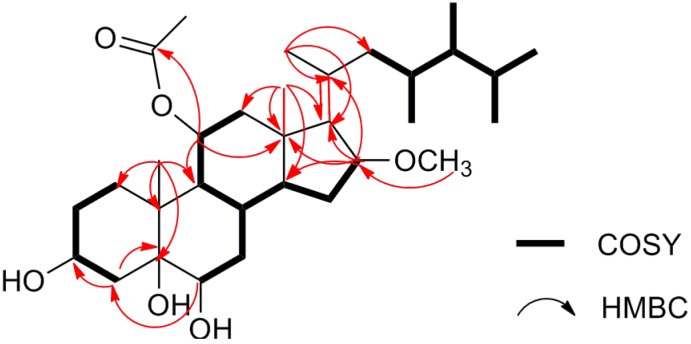
^1^H, ^1^H-COSY and selected HMBC correlations of compound **1**.

Compound **1** was isolated as a colorless amorphous solid with molecular formula of C_32_H_54_O_6_ established by HR-ESI-MS, which showed a pseudo-molecular-ion peak at *m/z* 557.3805 ((M + Na)^+^ C_32_H_54_O_6_Na^+^; cacld. 557.3813), requiring six degrees of unsaturation. The IR absorption bands at 3427 and 1710 cm^−1^ indicated the presence of OH and C=O groups, respectively, in agreement with the observations of the ^13^C NMR signals for four oxygenated C-atoms (δ_C_ 67.1, d; 71.0, d; 76.0, d and 76.6, s) and one C=O (δ_C_ 170.6, s). Its ^13^C NMR and DEPT spectra exhibited a total of 32 carbon signals classified into nine methyls (including one methoxyl), seven methylenes, ten methines (including four oxymethines), and six quaternary carbons (including one acetyl carbonyl and two olefinic carbons). These data together with the characteristic steroidal methyl signals at δ_H_ 0.85 (3H, d, *J* = 6.6 Hz, Me-26), 0.89 (3H, d, *J* = 6.6 Hz, Me-27), 0.78 (3H, d, *J* = 6.6 Hz, Me-28), 0.67 (3H, d, *J* = 6.6 Hz, Me-29), 0.92 (3H, s, Me-18), and 1.29 (3H, s, Me-19) (Table 1), strongly suggested compound **1** to be a polyhydroxylated C29-steroid, similar to the nucleus of sarcsteroids A and B [7] and to the side chain of 23,24-dimethylcholesta-17(20)-en-3β,5α,6β-triol [6]. This speculation was supported by ^1^H,^1^H-COSY and HMBC spectra as shown in Figure 2 (Supplementary Figures S9 and S11). The COSY correlations (H_2_-1/H_2_-2/H-3/H_2_-4, H-6/H_2_-7/H-8/H-9/H-11/H-12 and H-8/H-14/H_2_-15/H-16), together with the key HMBC correlations of Me-19 with C-1 (δ_C_ 33.3), C-5 (δ_C_ 76.6), C-9 (δ_C_ 48.7) and C-10 (δ_C_ 39.9), Me-18 with C-12 (δ_C_ 43.7), C-13 (δ_C_ 43.9), C-14 (δ_C_ 51.0) and C-17 (δ_C_ 144.7), and H-16 (δ_H_ 4.08 (d, *J* = 5.0 Hz)) with C-13 and C-17 could establish the 3,5,6-trihydroxy steroidal nucleus. HMBC correlations of H_3_-OAc (δ_H_ 1.99 (s)) and H-11 (δ_H_ 5.16 (dt, *J* = 12.1, 5.5 Hz)) with the carbonyl carbon signal (δ_C_ 170.6 s), and H_3_-OMe with C-16 (δ_C_ 81.7) positioned the OAc and OMe groups at C-11 and C-16, respectively. The consecutive COSY corrections starting from H_2_-22 (δ_H_ 2.07, 1.85) to H_3_-29, together with HMBC correlations (Figure 2) of H_3_-21 with C-17, C-20 (δ_C_ 135.0) and C-22 (δ_C_ 41.9) further indicated the presence of a 23,24-dimethylcholesterol side chain connected to the mother nucleus through a double bond between C-17 and C-20. The planar structure of compound **1** was thus depicted as shown in Figure 1.

**Table 1:** ^1^H and ^13^C NMR data (*J* and W_1/2_ values in Hz, δ in ppm) of compounds **1** and **3** in CDCl_3_ and **2** in C_5_D_5_N, *J* in Hz ^a^.

NO.	1			2			3		
	δ_H_	δ_C_		δ_H_	δ_C_		δ_H_	δ_C_	
1	1.18 m, 1.79 m	33.3	t	2.97 m, 2.76 m	35.4	t	3.63 br. s (W_1/2_ 8.8)	77.5	d
2	1.51 m, 1.81 m	31.2	t	2.22 m, 2.37 m	33.1	t	2.17 m, 1.82 m	37.0	t
3	4.06 m (W_1/2_ 24.0)	67.1	d	4.92 m (W_1/2_ 24.4)	67.4	d	4.36 m (W_1/2_ 22.0)	63.8	d
4	1.62 m, 2.07 m	41.1	t	2.41 m, 3.06 m	44.0	t	1.78 m, 2.09 m	41.3	t
5	−	76.6	s	−	76.5	s	−	77.2	s
6	3.56 br. s (W_1/2_ 7.1)	76.0	d	4.21 br. s	76.8	d	3.51 br. s	74.7	d
7	1.88 m, 1.68 m	34.7	t	2.03 m, 2.44 m	35.8	t	1.57 m, 1.91 m	34.6	t
8	1.94 m	28.1	d	2.33 m	30.1	d	1.91 m	29.5	d
9	1.81 m	48.7	d	2.35 m	53.1	d	2.21 m	43.9	d
10	−	39.9	s	−	41.1	s	−	42.3	s
11	5.16 dt (12.1, 5.5)	71.0	d	4.38 m	68.5	d	5.18 dt (10.8, 5.3)	72.4	D
12	2.65 dd (12.1, 5.5), 1.50 m	43.7	t	1.70 m, 2.71 m	53.0	t	2.43 dd (12.1, 5.3), 1.19 m	46.0	t
13	−	43.9	s	−	43.5	s	−	42.9	s
14	1.79 m	51.0	d	1.42 m	55.9	d	1.26 m	54.7	d
15	1.69 m, 1.21 m	29.4	t	1.21 m, 1.73 m	25.0	t	1.10 m, 1.64 m	24.1	t
16	4.08 d (5.0)	81.7	d	1.37 m, 2.05 m	28.6	t	1.91 m, 1.31 m	28.2	t
17	−	144.7	s	1.33 m	58.4	d	1.17 m	56.0	d
18	0.92 s	16.7	q	0.81 s	13.4	q	0.74 s	12.7	q
19	1.29 s	17.4	q	1.93 s	17.7	q	1.20 s	16.5	q
20	−	135.0	s	1.04 m	35.6	d	1.38 m	35.4	d
21	1.70 s	18.1	q	1.09 d (6.6)	21.3	q	0.91 d (6.4)	18.6	q
22	2.07 m, 1.85 m	41.9	t	0.28 m	32.6	d	1.40 m, 1.04 m	35.9	t
23	1.81 m	32.4	d	−	25.0	s	1.86 m, 2.02 m	24.7	t
24	1.05 m	44.2	d	1.34 m	49.7	d	5.07 t (7.4)	125.0	d
25	1.57 m	30.3	d	−	149.3	s		131.1	s
26	0.85 d (6.6)	22.0	q	4.87 s, 4.76 s	110.3	t	1.59 s	17.7	q
27	0.89 d (6.6)	19.6	q	1.77 s	23.6	q	1.68 s	25.7	q
28	0.78 d (6.6)	11.5	q	1.09 d (6.6)	16.1	q	−	−	
29	0.67 d (6.6)	13.5	q	0.85 s	15.2	q	−	−	
30	−			0.55 dd (9.1, 4.1), −0.13 dd (5.7, 4.4)	20.0	t	−	−	
Ac	1.99 s	21.6	q	−	−		2.04 s	21.7	q
		170.6	s	−	−		−	169.3	s
OMe	3.27 s	56.3	q	−	−		−	−	

^a^^,^^1^H and ^13^C NMR at 600 and 150 MHz, respectively.

**Figure 3:**
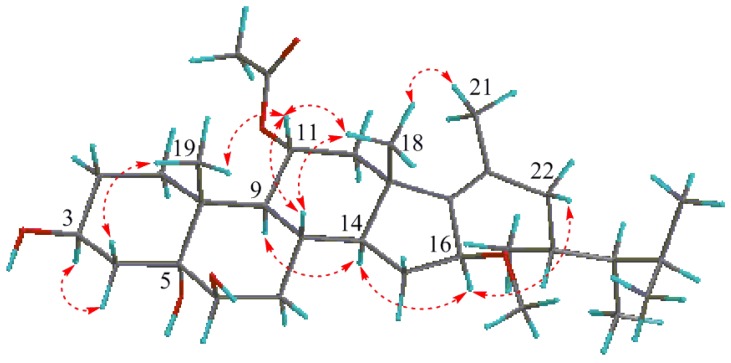
Key NOESY correlations for compound **1**.

The relative configuration of the stereogenic centers for **1** was deduced from the proton-proton coupling constants (Table 1) and NOESY spectrum (Figure 3 and Supplementary Figure S12). The half-peak-width of H-6 (δ_H_ 3.56 (br. s, W_1/2_ = 7.1 Hz)) was indicative of its equatorial α-orientation, while the half-width of about 20.0 Hz for H-3 and the large coupling constants for H-11 (dd, *J* = 12.1, 5.5 Hz) pointed to their axial α- and β-orientations, respectively [8]. The NOESY cross peaks between Me-19 and Hβ-4 (δ_H_ 2.07), H-11 and H-8, between H-11 and Me-18, and between H-14 and H-9, H-16 and H-14 indicated their special relative orientations as shown in Figure 3. The orientations of the OH groups, especially the tertiary OH group at C-5, were assigned by the pyridine-induced deshielding effect. The marked downfield shifts of H-3α and H_3_-19β in pyridine-*d*_5_ (δ_H-3_ 4.86, δ_Me-19_ 1.83) relative to CDCl_3_ (δ_H-3_ 4.06, δ_Me-19_ 1.29) which was attributed to the 1,3-*syn*-periplanar arrangement of hydroxyl groups (1,3 diaxial deshieding effect) were observed, indicating the orientations of OH-5α and OH-6β [9]. The *Z* geometry of the double bond Δ^17^^(20)^ was deduced from the NOESY cross peaks between Me-18 and Me-21, and between H-16 and H_2_-22. In addition, the NOESY correlation of H-16 with H-14 but the lack of H-16 with H-18 indicated the α-orientation of H-16. Tentative assignments of 23*R* and 24*R* for the side chain of **1** were evident from the almost consistent ^13^C NMR data with the reported (23*R*,24*R*)-23,24-dimethylcholesta-17(20)-en-3β,5α,6β-triol [6]. Accordingly, compound **1** was determined as (23*R*,24*R*,17*Z*)-11α-acetoxy-16β-methoxy-23,24-dimethylcholest-17(20)-en-3β,5α,6β-triol, which was suspected to be an artifact from the isolation process.

The molecular formula of compound **2** was determined as C_30_H_50_O_4_ from the HR-ESI-MS data ((M + Na)^+^ 497.3614; calcd. 497.3601). In its 1D NMR spectra, six methyl proton signals (δ_H_ 0.81 (3H, s), 0.85 (3H, s), 1.93 (3H, s), 1.09 (6H, d, *J* = 6.6 Hz), 1.77 (3H, s)) and four oxygenated carbon signals (δ_C_ 67.4 (d), 68.5 (d), 76.5 (s), 76.8 (d)), as well as a characteristic cyclopropane-bearing gorgosterol-type side chain (δ_H_ −0.13 (1H, dd, *J* = 5.7, 4.4 Hz, H-30a), 0.28 (1H, m, H-22), 0.55 (1H, dd, *J* = 9.1, 4.1 Hz, H-30b)) suggested **2** as a polyhydroxygorgosterol. Careful comparison the NMR data of **2** with those of the reported sarcoaldesterol A [10] showed their closely structural similarity except that the isopropenyl group in side chain of **2** replaced the isopropyl group in sarcoaldesterol A. The HMBC correlations (Supplementary Figure S20) of H-26 (δ_H_ 4.76, 4.87 (each 1H, s)) with C-24 (δ_C_ 49.7 (d)), C-25 (δ_C_ 149.3 (s)) and C-27 (δ_C_ 23.6 (q)) supported the above assumption. The relative configuration of **2** was assigned to be the same as sarcoaldesterol A, evidenced from NOESY spectrum (Supplementary Figure S21) and similar NMR data together with biogenetic considerations. The structure of **2** was evidently established as (24*R*)-gorgost-25-en-3β,5α,6β,11α-tetraol.

Molecular formula C_29_H_48_O_6_ was assigned to compound **3** deduced from HR-ESI-MS at *m*/*z* 515.3357 ((M + Na)^+^). It was similar to sarcsteroid B according to their ^1^H and ^13^C NMR spectra [6,7]. The marked difference was the presence of an additional OH group at C-1 in **3**, which was confirmed by an oxygenated carbon signal (δ_C_ 77.5 (d, C-1)), a proton signal (δ_H_ 3.63 (br s, H-1)) and HMBC correlation (Supplementary Figure S29) of H_3_-19 (δ_H_ 1.20 (s)) with C-1. The half-peak-width of H-1 (br. s, W_1/2_ = 8.8 Hz) and NOESY cross peak (Supplementary Figure S30) between Me-19 and H-1 were indicative of its equatorial β-orientation. Thus, compound **3** was identified as 11α-acetoxycholest-24-en-1α,3β,5α,6β-tetraol.

Seven known analogs (Figure 1), (24*R*)-methylcholest-7-en-3β,5α,6β-triol (**4**) [11], 11α-acetoxy-cholest-24-en-3β,5α,6β-triol (**5**) [6,7], (22*E*,24*S*)-11α-acetoxy-ergost-22,25-dien-3β,5α,6β-triol (**6**) [6,7], (24*S*)-11α-acetoxy-ergost-3β,5α,6β-triol (**7**) [12], (24*R*)-11α-acetoxy-gorgost-3β,5α,6β-triol (**8**) [6,7], (24*S*)-ergost-3β,5α,6β, 11α-tetraol (**9**) [10] and (24*S*)-23,24-dimethylcholest-22-en-3β,5α,6β,11α-tetraol (**10**) [6,7] were also isolated and identified by comparison of their spectroscopic data with those reported in the literature.

Because of the cytotoxicities [13,14] and antiviral activities [15] for some reported polyhydroxylated steroids, the cytotoxicities for all the compounds (**1**–**10**) against three tumor cell lines (human leukemia, K562; human myeloid leukemia, HL-60; human cervical carcinoma, HeLa) were evaluated *in vitro* by MTT (3-(4,5-dimethylthiazol-2-yl)2,5-diphenyl-2H-tetrazolium bromide) and SRB (Sulforhodamine B) methods (Table 2). Compounds **1**, **3**, and **5**–**8**, which shared the same nucleus substituted by an OAc group at C-11, showed potent cytotoxicities against certain cell lines with IC_50_ values less than 10.3 μM, while compounds **4** without substitution, **9** and **10** with OH substitution at C-11 were inactive against tested cell lines, indicating the 11-OAc group to be a very important pharmacophore. Compound **2** with an OH group at C-11 showed selective inhibitory activity only against K562 cell line with IC_50_ value of 9.9 μM, compounds **9** and **10** with the same substitution also showed the similar selectivity, implying that the OH substitution at C-11 could improve the selectivity but decrease activity. Meanwhile, compounds **1**, **6**, and **8** showed broad-spectrum activities against three cell lines. The comparative analysis of the cytotoxic data for compounds **3** and **5** indicated that the 1-OH substitution did not contribute to the activity.

**Table 2:** Cytotoxicity data (IC_50_ in μM) for compounds **1**–**10**.

Compounds	K562 ^a^	HL-60 ^a^	HeLa ^b^
**1**	17.3	9.3	17.0
**2**	9.9	>50	>50
**3**	10.1	17.2	>50
**4**	21.7	33.7	>50
**5**	9.1	14.3	>50
**6**	6.4	10.5	11.5
**7**	10.3	12.8	>50
**8**	9.8	14.6	24.7
**9**	24.5	32.5	>50
**10**	26.6	>50	>50
ADM(Adriamycin) ^c^	0.2	0.02	0.6

^a^: By MTT method; ^b^: By SRB method; ^c^: Positive control.

Antiviral activities of all the isolates against H1N1 IAV were also evaluated by the cytopathic effects assays (CPE). Only compounds **4** and **9**, despite of their lack of cytotoxicity in the above experiments, exhibited significant anti-H1N1 virus activity with IC_50_ values of 19.6 and 36.7 μg/mL (Ribavirin was used as a positive control with IC_50_ value of 24.6 μg/mL), respectively. This is the first report of a specific type of steroid having activity against a flu virus, except for the one report that a similar type of steroid had activity against human cytomegalovirus [15].

## 3. Experimental Section

### 3.1. General Methods

Optical rotations were measured with a JASCO P-1020 polarimeter (JASCO Corporation, Tokoyo, Japan). UV spectra were measured with a Beckman DU640 spectrophotometer (Beckman Coulter Inc., Brea, CA, USA). IR spectra were recorded on a Nicolet NEXUS 470 spectrophotometer (International Equipment Trading Ltd., Vernon Hills, IL, USA). NMR spectra were measured with a JEOL JNMECP 600 (JEOL Ltd., Tokoyo, Japan) and Bruker 600 spectrometers (Bruker Daltonics Inc., Billerica, MA, USA). The melting points uncorrected were measured on an X-4 micro-scopic melting point apparatus (Shanghai Instrument Physical Optics Instrument Co. Ltd., Shanghai, China). Chemical shifts were referenced to residual non deuterated solvent signals (CDCl_3_: δ_H_ 7.26 ppm, δ_C_ 77.0 ppm; C_5_D_5_N: δ_H_ 7.21, 7.58, 8.73 ppm, δ_C_ 123.5, 135.5, 149.9 ppm). HR-ESI-MS data were obtained on a Micromass Q-Tof Ultima GLOBAL GAA076 LC mass spectrometer on a Thermo Scientific LTQ orbitrap XL mass spectrometer (Thermo Fisher Scientific Inc., Waltham, MA, USA). HPLC isolation was achieved on a Waters 2695 instrument using a semi-preparative HPLC column (YMC-packed C18, 5 μm, 250 × 10 mm) (YMC Co. Ltd., Kyoto, Japan). Vacuum liquid chromatography (VLC) was performed on special chromatographic column utilizing reduced pressure to increase the flow rate of the mobile phase. Silica-gel (200–300 mesh, 300–400 mesh, Qingdao Marine Chemical Factory, Qingdao, Shandong, China) and ODS silica-gel (50 μm, Merck, Darmstadt, Germany) were used for column chromatography (CC). TLC was carried out with glass precoated silica gel GF_254_ plates (Qingdao Marine Chemical Factory, Qingdao, Shandong, China). Spots were visualized under UV light or by spraying with 10% H_2_SO_4_ in EtOH-H_2_O (95:5, v/v) followed by heating.

### 3.2. Animal Material

The *Sarcophyton* sp. was collected from the South Sea (Weizhou Islands sea area) at a depth of 12 m. The specimen was identified by Professor Zou, R.L. (South China Sea Institute of Oceanology, Chinese Academy of Sciences, Guangzhou, China). The voucher specimen (NO. WZD-2010-03) was deposited at State Key Laboratory of Marine Drugs, Ocean University of China, Qingdao, Shandong, China.

### 3.3. Extraction and Isolation

The frozen sample of *Sarcophyton* sp. (5.0 kg, wet weight) was homogenized and then extracted with MeOH for three times (5 L × 3, each, 3 days) at room temperature, and the solution was evaporated in vacuum to yield a crude extract (70.0 g) which was subjected to column chromatography (CC) on silica gel using petroleum ether/acetone (from 100:1 to 1:2, v/v) as eluent to obtain seven fractions (Fr. 1–Fr. 7). Each fraction was detected by TLC and was preliminarily bio-assayed for cytotoxicities, and Fr. 6 was found to be the most active fraction containing the main polyhydroxylated steroids of this species. Thus, Fr. 6 (4.58 g) was subjected to Sephadex LH-20 (MeOH) and was further separated by repeat chromatography on a silica gel column eluting with petroleum ether/acetone (from 3:1 to 1:1) to afford four sub-fractions (Fr. 6.1–Fr. 6.4). Fr. 6.2 (68.1 mg) was purified by ODS column chromatograph with a gradient increasing MeOH in H_2_O (from 30% to 85%) to yield Fr. 6.2.1–Fr. 6.2.3. Fr. 6.2.3 (30 mg) was further separated by HPLC (ODS. C18; 90% MeOH in H_2_O) to yield compound **4** (2.8 mg). Fr. 6.3 (558.5 mg) was applied to an ODS column and eluted with MeOH in H_2_O (from 30% to 85%) to obtain Fr. 6.3.1–Fr. 6.3.4. Fr. 6.3.2 (61.4 mg) was further purified by HPLC (ODS. C8; 80% MeOH in H_2_O) to yield compound **5** (4.1 mg) and compound **6** (2.2 mg). Fr. 6.3.3 (255.1 mg) was further separated to three fractions (Fr. 6.3.3.1–Fr. 6.3.3.3) by Sephadex LH-20 (petroleum ether/CH_2_Cl_2_/MeOH 2:2:1). Fr. 6.3.3.1 (29.1 mg) was repurified with HPLC (ODS. C18; 83% MeOH in H_2_O with 1‰ HCOOH) to afford compound **7** (4.9 mg). Fr. 6.3.4 (105.6 mg) was also purified by HPLC (ODS. C8; 85% MeOH in H_2_O with 1‰ HCOOH) to yield compound **8** (37.3 mg). Fr. 6.4 (1.0 g) was submitted to a silica gel column eluting with CH_2_Cl_2_/MeOH/H_2_O (300:10:3.3) to afford two fractions (Fr. 6.4.1 and Fr. 6.4.2). Fr. 6.4.1 (109.1 mg) was further separated by HPLC (ODS. C18; 77% MeOH in H_2_O) to yield compound **3** (4.2 mg) and Fr. 6.4.2 (33.2 mg) was purified by HPLC (ODS. C8; 80% MeOH in H_2_O with 1‰ HCOOH) to yield compounds **1** (3 mg), **2** (5.1 mg), **9** (1.9 mg) and **10** (8.7 mg).

*Compound* **1**: (23*R*,24*R*,17*Z*)-11α-acetoxy-16β**-**methoxy-23,24-dimethylcholest-17(20)-en-3β,5α,6β-triol. Colorless amorphous solid; melting point (m.p.) 234–236 °C; 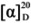 = −67.1 (*c* = 2.7, CHCl_3_); IR (KBr): 3427, 2960, 2873, 1731, 1710, 1377, 1250, 1068, 1025 cm^−1^; ^1^H and ^13^C NMR: see Table 1; HR-ESI-MS: *m/z* 557.3805 [M + Na]^+^ (calcd. for C_32_H_54_NaO_6_^+^, 557.3813).

*Compound* **2**: (24*R*)-gorgost-25-en-3β,5α,6β,11α-tetraol. Colorless amorphous solid; m.p. 261–262 °C; 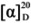 = −2.69 (*c* = 3.2, MeOH); IR (KBr): 3422, 2925, 1702, 1459, 1378, 1261, 1097, 1032 cm^−1^; ^1^H and ^13^C NMR: see Table 1; HR-ESI-MS: 497.3614 [M + Na]^+^ (calcd. for C_30_H_50_NaO_4_^+^, 497.3601).

*Compound* **3**: 11α-acetoxycholest-24-en-1α,3β,5α,6β-tetraol. Colorless amorphous solid; m.p. 266–268 °C; 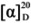 = −17.82 (*c* = 3.9, CHCl_3_); IR (KBr): 3394, 2926, 2889, 1727, 1378, 1257, 1068, 1040 cm^−1^; ^1^H and ^13^C NMR: see Table 1; HR-ESI-MS: 515.3357 [M + Na]^+^ (calcd. for C_29_H_48_NaO_6_^+^, 515.3343).

### 3.4. Cytotoxic Assay

*In vitro* cytotoxicities were determined by MTT [3-(4,5-dimethylthiazol-2-yl)-2,5-diphenyltetrazolium bromide] colorimetric assay [16] against K562 (human leukemia cells) and HL-60 (human myeloid leukemia cells), SRB (Sulforhodamine B) assay [17] against HeLa (human cervical carcinoma cells). All the cell lines were purchased from Shanghai Institute of Cell Biology (Shanghai, China). Cytotoxic data (Table 2) for compounds **1**–**10** were obtained on the basis of five concentrations with three replications. Adriamycin (doxorubicin, ADM) was used as a positive control, and IC_50_ values >50 μM were considered to be inactive in cytotoxic assays.

In the MTT assay, the cells were cultured in RPMI-1640 supplemented with 10% FBS under a humidified atmosphere of 5% CO_2_ and 95% air at 37 °C. Those cell suspensions (200 μL) at a density of 5 × 104 cells/mL were plated in 96-well microtiter plates and incubated for 24 h at the above conditions. The test compound solution (2 μL in DMSO) at different concentrations in triplicate was added to each well and further incubated for 72 h under the same conditions. 20 μL of the MTT solution (5 mg/mL in IPMI-1640 medium) was then added to each well and incubated for 4 h. The old medium containing MTT (150 μL) was then gently replaced by DMSO and vibrated to dissolve any formazan [1-(4-Iodophenyl)-5-(4-nitrophenyl)-3-phenylformazan] crystals formed. The optical density of the solution was measured on a Spectra Max Plus plate reader at 570 nm. The IC_50_ value of each compound was calculated by Reed and Muench’s method.

In the SRB assay, 200 μL of the cell suspensions were plated in 96-well plates at a density of 2 × 10^5^ cell mL^−1^. Then 2 μL of the test solutions (in MeOH) was added to each well, and the culture was further incubated for 24 h. The cells were fixed with 12% trichloroacetic acid, and the cell layer was stained with 0.4% SRB. The absorbance of the SRB solution was measured at 515 nm. Dose-response curves were generated, and the IC_50_ values (the concentration of compound required to inhibit cell proliferation by 50%) were calculated from the linear portion of log dose-response curves.

### 3.5. Anti-H1N1 Virus Assay

The antiviral activity against H1N1 was evaluated by the CPE inhibition assay [18]. Confluent MDCK cell monolayers were firstly incubated with influenza virus (A/Puerto Rico/8/34 (H1N1), PR/8) at 37 °C for 1 h. After removing the virus dilution, cells were maintained in infecting media (RPMI 1640, 4 μg/mL of trypsin) containing different concentrations of test compounds at 37 °C. After 48 h incubation at 37 °C, cells were fixed with 100 μL of 4% formaldehyde for 20 min at room temperature. After removal of the formaldehyde, the cells were stained with 0.1% crystal violet for 30 min. The plates were washed and dried, and the intensity of crystal violet staining for each well was measured in a microplate reader (Bio-Rad, Hercules, CA, USA) at 570 nm. The IC_50_ was calculated as the compound concentration required inhibiting CPE production at 48 h post-infection by 50%. Ribavirin (LuKang Cisen (Jining, China)) was used as positive control, and compounds with an inhibition rate of >70%, >50%, and <30% at 50 μg/mL were respectively regarded having strong, moderate, and weak activities.

## 4. Conclusions

Polyhydroxylated steroids are widely distributed in Alcyonarian corals of the genera *Sarcophyton*, *Sinularia*, and *Lobophytum*. The present work offered three new members of 3β,5α,6β-triol steroidal clusters and firstly reported the preliminary structure-activity relationships for cytotoxicities and anti-H1N1 virus activities of the isolates. As a consequence, the OAc group at C-11 in those steroids was considered as an important pharmacophore for their cytotoxicities.

## Supplementary Files

Supplementary File 1 Supplementary Information (PDF, 4789 KB)
