# Supplementary material for: Polyhydroxylated Steroids from the South China Sea Soft Coral Sarcophyton sp. and Their Cytotoxic and Antiviral Activities
Source: Mar Drugs. 2013 Dec 2;11(12):4788–98. doi: 10.3390/md11124788 (PMC3877887; doi:10.3390/md11124788)

Supplementary Information

Table S1. Inhibition rates of compounds **1**–**10** in preliminary cytotoxicity test.
Table S2. Inhibition rates of compounds with anti-H1N1 virus activities in preliminary test.
Figure S1. Animal material: The *sarcophyton* sp. was collected from the South Sea (Weizhou Islands sea area, Guangxi, China) at a depth of 12 m. The specimen was identified by Professor Zou, R.L. (South China Sea Institute of Oceanology, Chinese Academy of Sciences, Guangzhou, China). The voucher specimen (NO. WZD-2010-03) was deposited at State Key Laboratory of Marine Drugs, Ocean University of China, Qingdao, China.
Figure S2. The positive HRESIMS spectrum of compound (**1**).
Figure S3. ^{1}H-NMR (600 MHz, $CDCl_3$) spectrum of compound (**1**).
Figure S4. The amplificatory ^{1}H NMR (600 MHz, $CDCl_3$) spectrum of compound (**1**).
Figure S5. ^{1}H-NMR (600 MHz, C_5D_5N) spectrum of compound (**1**).
Figure S6. The amplificatory ^{1}H NMR (600 MHz, C_5D_5N) spectrum of compound (**1**).
Figure S7. ^{13}C-NMR (150 MHz, $CDCl_3$) spectrum of compound (**1**).
Figure S8. DEPT (150 MHz, $CDCl_3$) spectrum of compound (**1**).
Figure S9. ^{1}H-^{1}H COSY spectrum of compound (**1**).
Figure S10. HMQC spectrum of compound (**1**).
Figure S11. HMBC spectrum of compound (**1**).
Figure S12. NOESY spectrum of compound (**1**).
Figure S13. The positive HRESIMS spectrum of compound (**2**).
Figure S14. ^{1}H-NMR (600 MHz, C_5D_5N) spectrum of compound (**2**).
Figure S15. The amplificatory ^{1}H NMR (600 MHz, C_5D_5N) spectrum of compound (**2**).
Figure S16. ^{13}C-NMR (150 MHz, C_5D_5N) spectrum of compound (**2**).
Figure S17. DEPT (150 MHz, C_5D_5N) spectrum of compound (**2**).
Figure S18. ^{1}H-^{1}H COSY spectrum of compound (**2**).
Figure S19. HMQC spectrum of compound (**2**).
Figure S20. HMBC spectrum of compound (**2**).
Figure S21 NOESY spectrum of compound (**2**).
Figure S22. The positive HRESIMS spectrum of compound (**3**).
Figure S23. ^{1}H-NMR (600 MHz, $CDCl_3$) spectrum of compound (**3**).
Figure S24. The amplificatory ^{1}H NMR (600 MHz, $CDCl_3$) spectrum of compound (**3**).
Figure S25. ^{13}C-NMR (150 MHz, $CDCl_3$) spectrum of compound (**3**).
Figure S26. DEPT (150 MHz, $CDCl_3$) spectrum of compound (**3**).
Figure S27. ^{1}H -^{1}H COSY spectrum of compound (**3**).
Figure S28. HMQC spectrum of compound (**3**).
Figure S29. HMBC spectrum of compound (**3**).
Figure S30. NOESY spectrum of compound (**3**).

Table S1. Inhibition rates of compounds **1**–**10** in preliminary cytotoxicity test.

	**K562**		**HL-60**		**HeLa**	
	**Inhibition Ratio (%)**	**OD Value**	**Inhibition Ratio (%)**	**OD Value**	**Inhibition Ratio (%)**	**OD Value**
Adramycin (1 µM)	80.17	0.31 ±0.00	85.85	0.18 ±0.00	54.89	0.84 ±0.02
**1** (50 µM)	91.43	0.13 ±0.00	55.49	0.57 ±0.06	47.33	0.96 ±0.04
**2** (50 µM)	89.99	0.15 ±0.01	25.72	0.95 ±0.03	46.45	0.98 ±0.09
**3** (50 µM)	91.07	0.13 ±0.00	50.65	0.63 ±0.02	64.35	0.65 ±0.05
**4** (50 µM)	76.23	0.36 ±0.00	42.79	0.73 ±0.01	-9.90	2.00 ±0.05
**5** (50 µM)	91.05	0.14 ±0.00	51.25	0.62 ±0.01	17.52	1.50 ±0.02
**6** (50 µM)	91.69	0.13 ±0.00	63.28	0.47 ±0.02	77.05	0.42 ±0.01
**7** (50 µM)	90.74	0.14 ±0.00	52.77	0.60 ±0.01	36.45	1.16 ±0.03
**8** (50 µM)	90.90	0.14 ±0.00	47.62	0.67 ±0.06	74.62	0.46 ±0.06
**9** (50 µM)	68.10	0.48 ±0.01	31.68	0.87 ±0.01	-1.24	1.84 ±0.07
**10** (50 µM)	65.31	0.52 ±0.02	28.96	0.91 ±0.02	28.33	1.31 ±0.06

Table S2. Inhibition rates of compounds with anti-H1N1 virus activities in preliminary test.

	**Concentration (µg/mL)**	**Inhibition Ratio (%)**
Ribavirin	50	72.4
**4**	50	54.3
**5**	50	30.5
**9**	50	52.7
**10**	50	48.6

Figure S1. Animal material: The *sarcophyton* sp. was collected from the South Sea (Weizhou Islands sea area, Guangxi, China) at a depth of 12 m. The specimen was identified by Professor Zou, R.L. (South China Sea Institute of Oceanology, Chinese Academy of Sciences, Guangzhou, China). The voucher specimen (NO. WZD-2010-03) was deposited at State Key Laboratory of Marine Drugs, Ocean University of China, Qingdao, China.

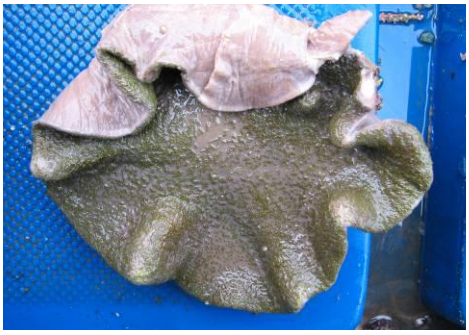

Sarcophyton sp.

Figure S2. The positive HRESIMS spectrum of compound (**1**).

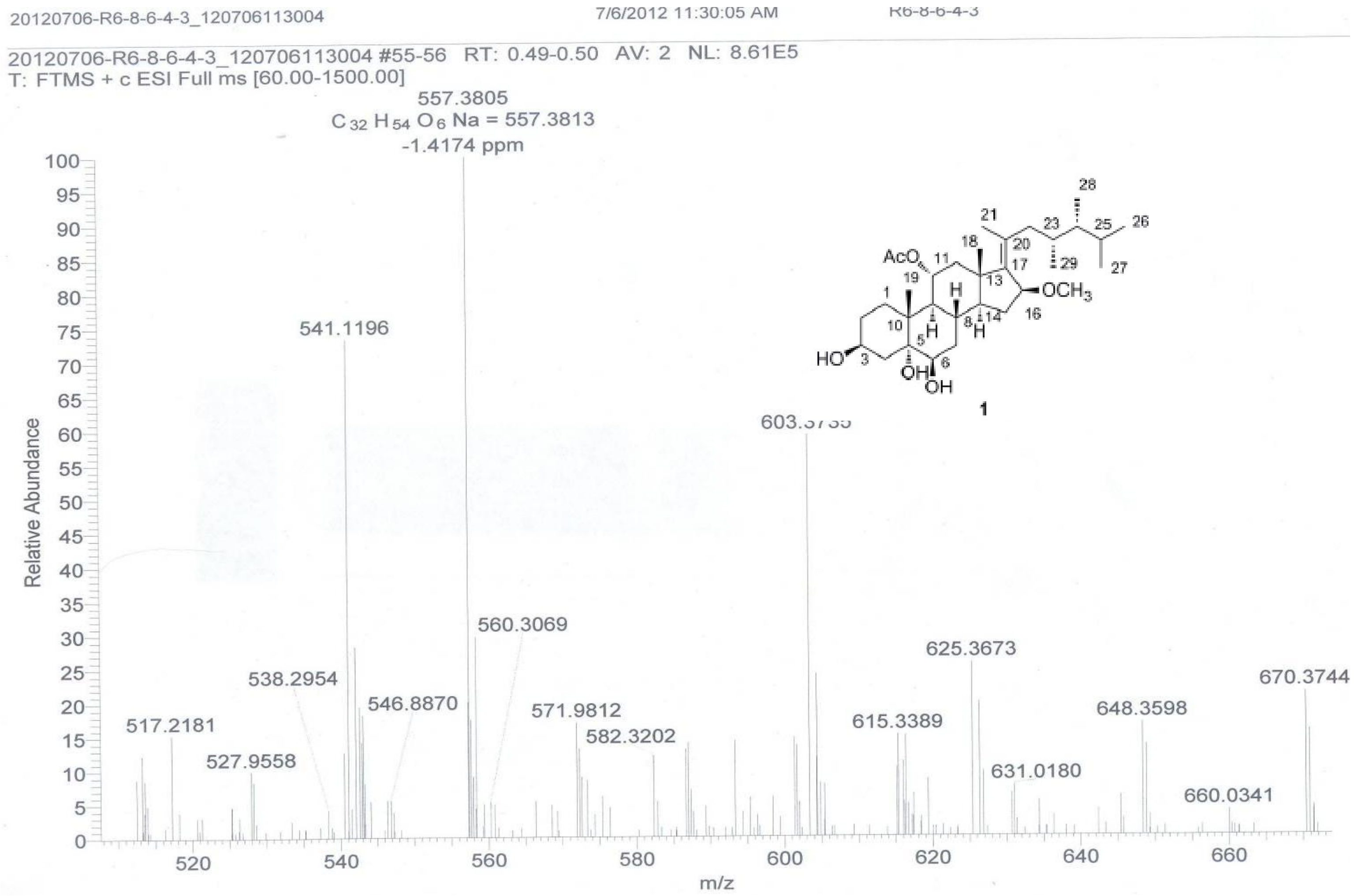

Figure S3. ^{1}H-NMR (600 MHz, $CDCl_3$) spectrum of compound (**1**).

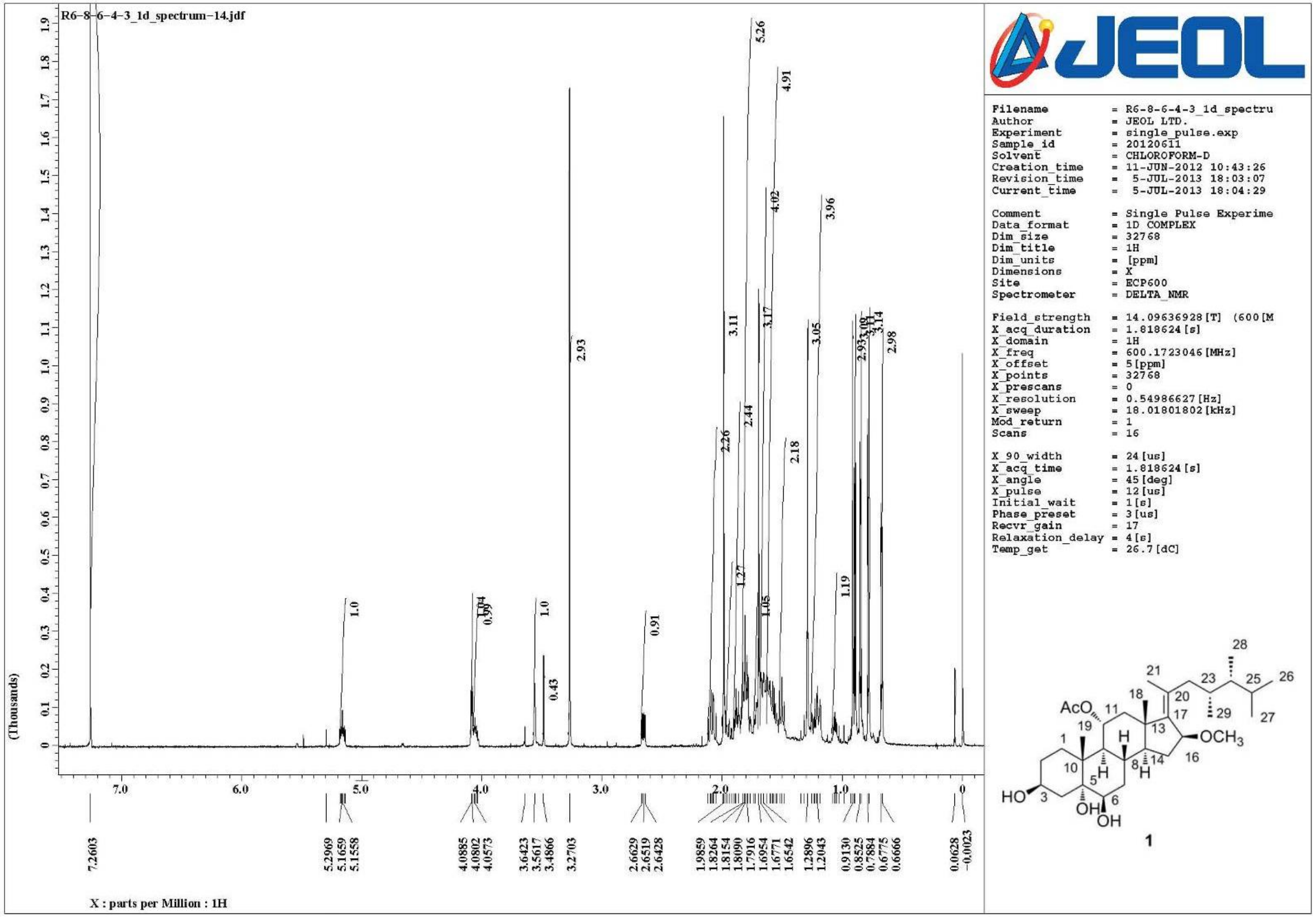

Figure S4. The amplificatory ^{1}H NMR (600 MHz, $CDCl_3$) spectrum of compound (**1**).

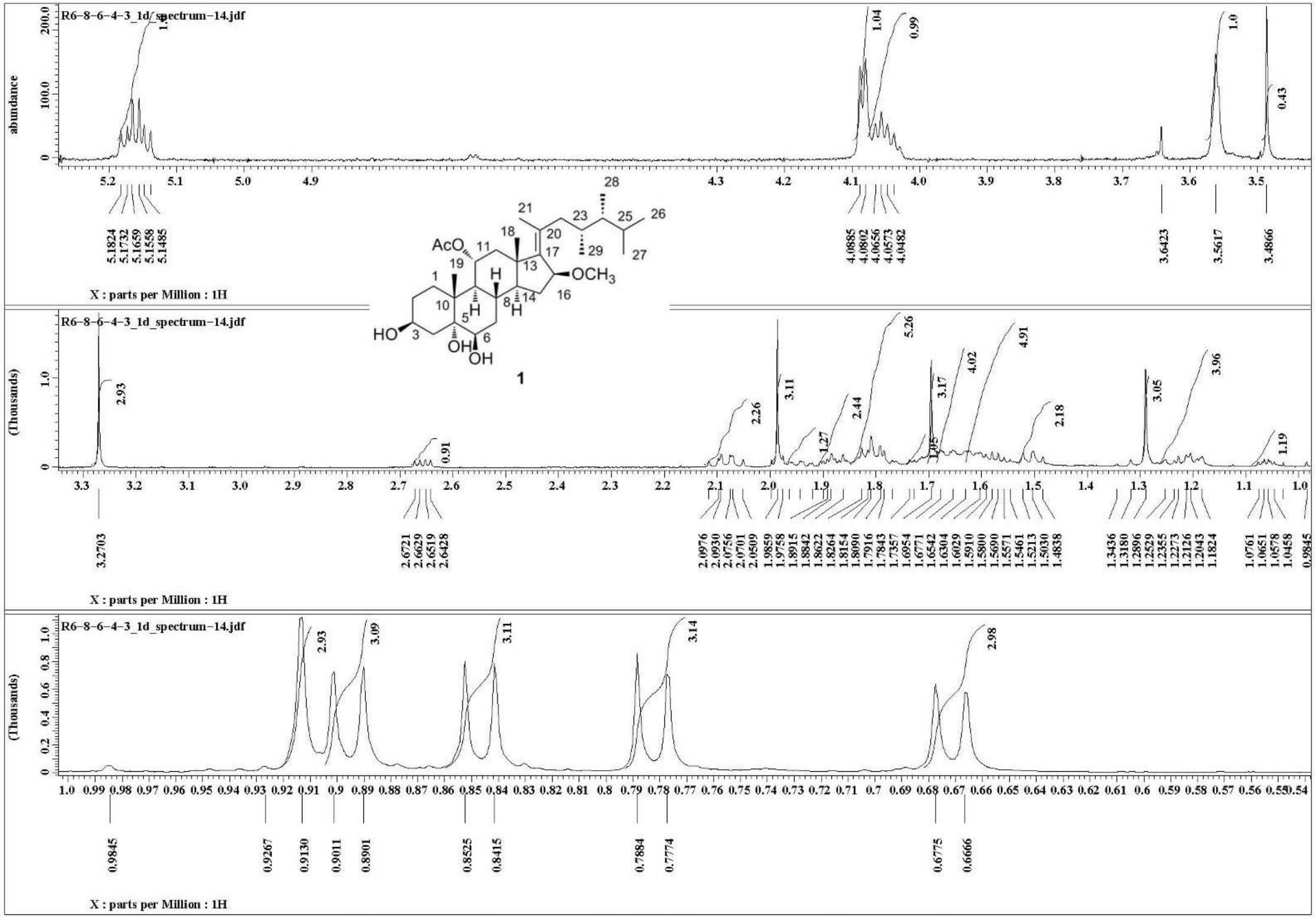

Figure S5. ^{1}H-NMR (600 MHz, C_5D_5N) spectrum of compound (**1**).

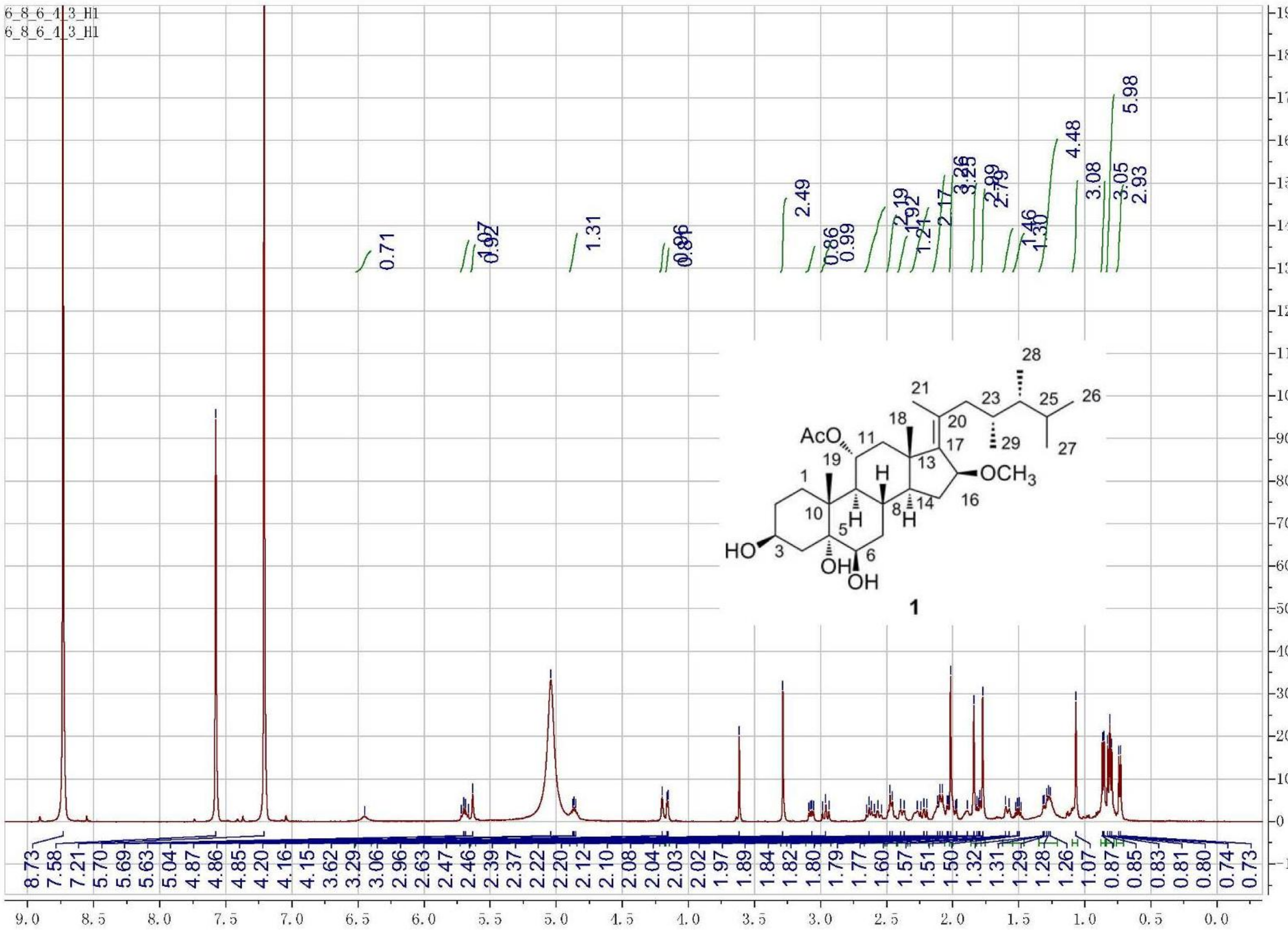

Figure S6. The amplificatory ^{1}H NMR (600 MHz, C_5D_5N) spectrum of compound (**1**).

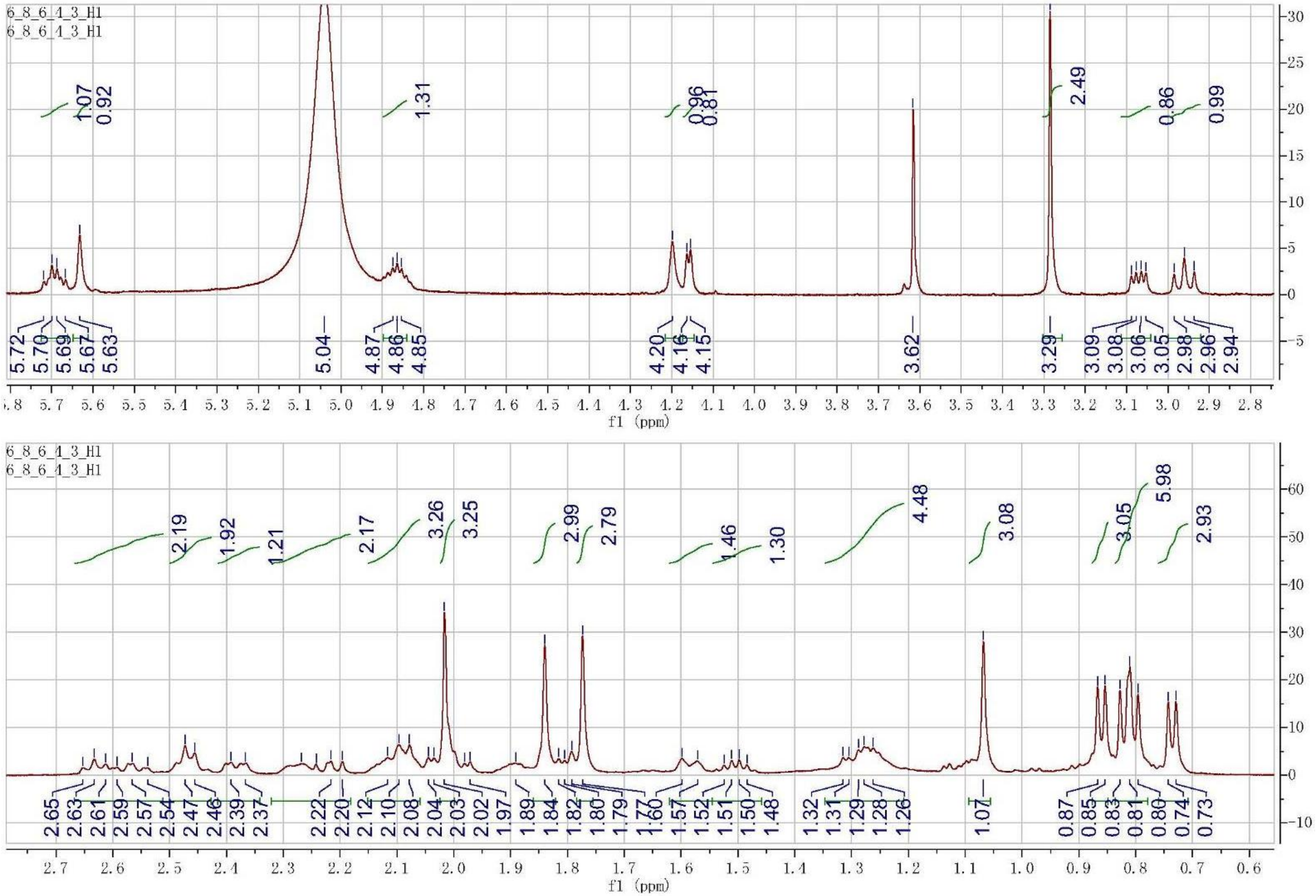

Figure S7. ^{13}C-NMR (150 MHz, $CDCl_3$) spectrum of compound (**1**).

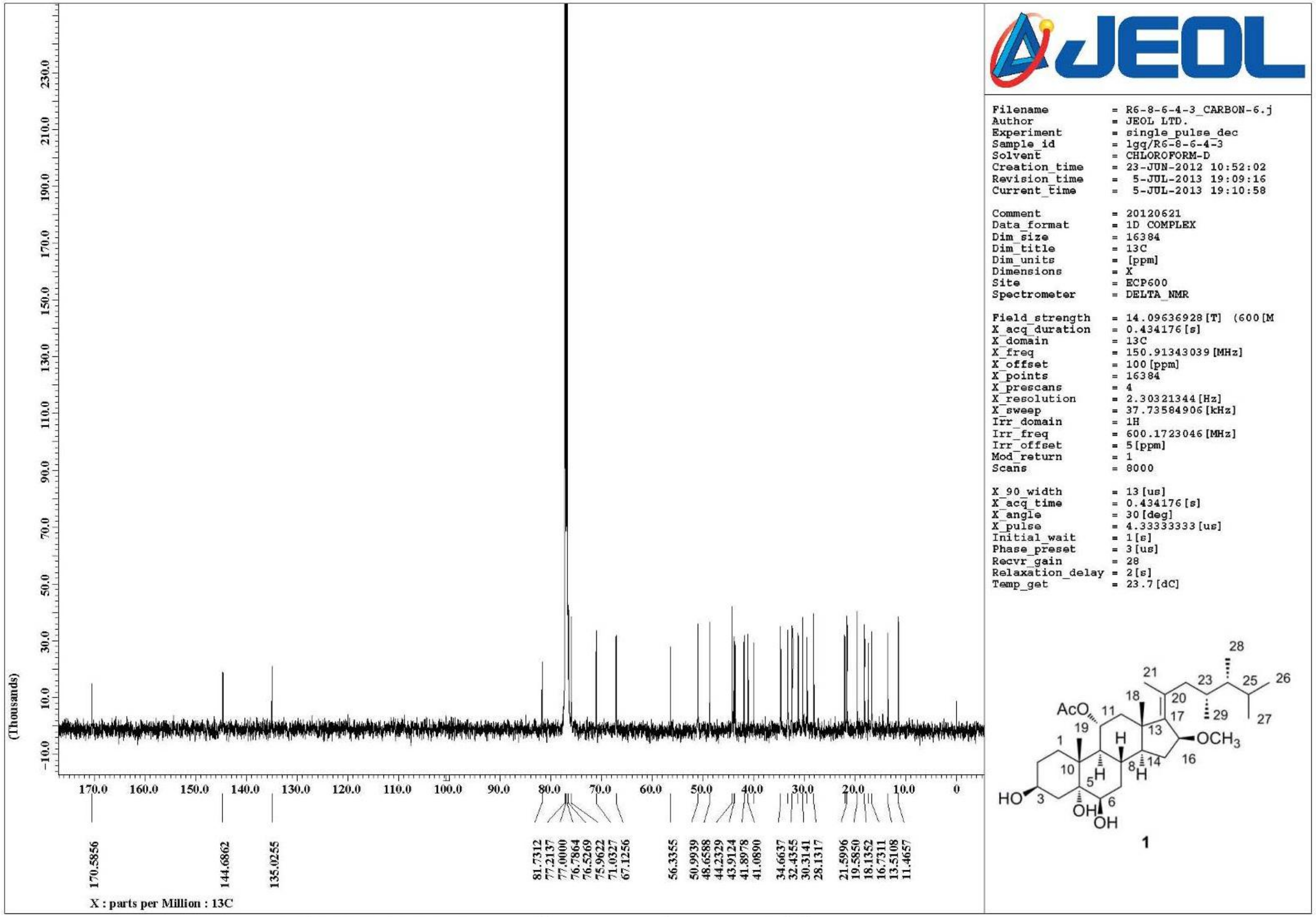

Figure S8. DEPT (150 MHz, $CDCl_3$) spectrum of compound (**1**).

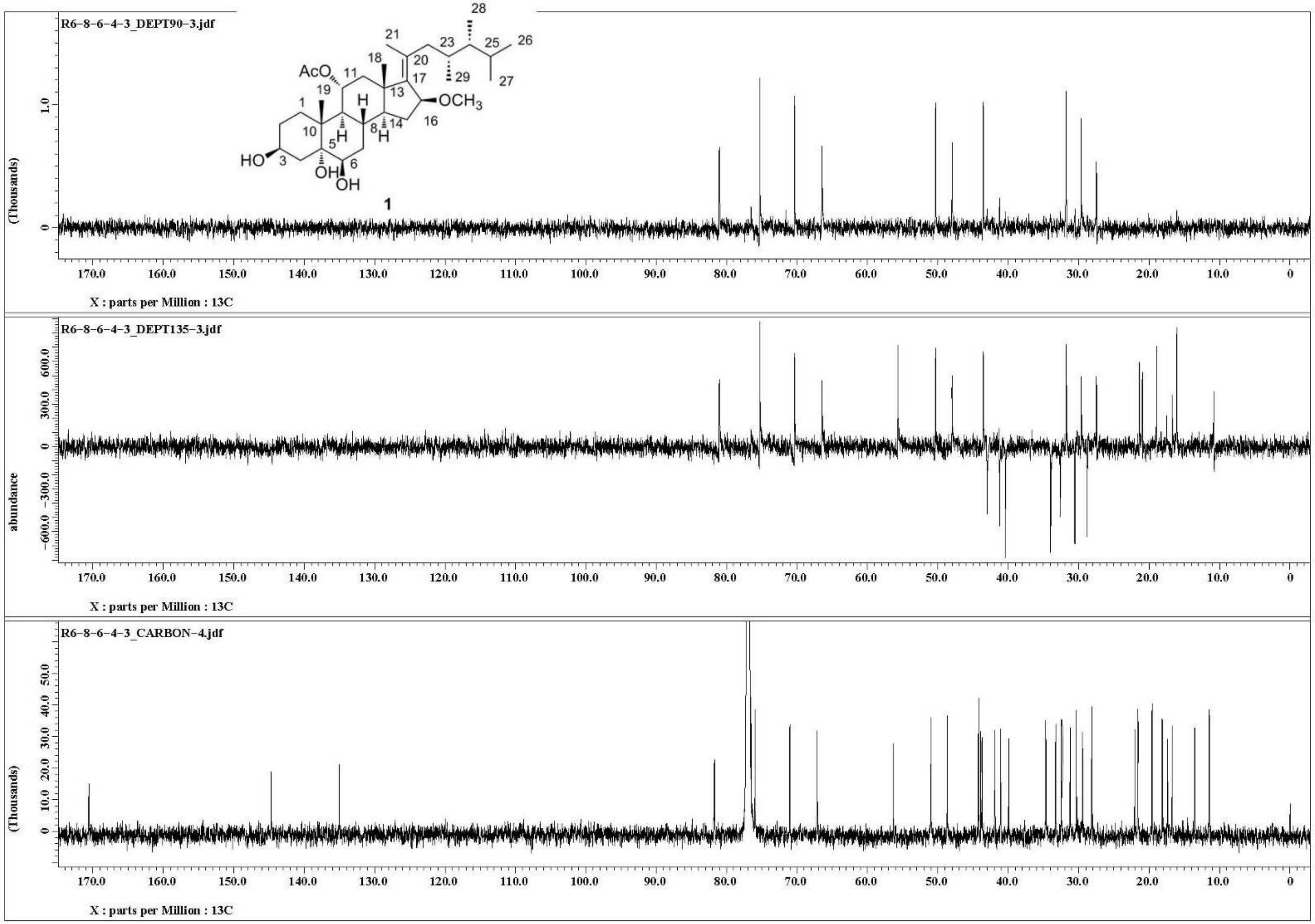

Figure S9. ^{1}H-^{1}H COSY spectrum of compound (**1**).

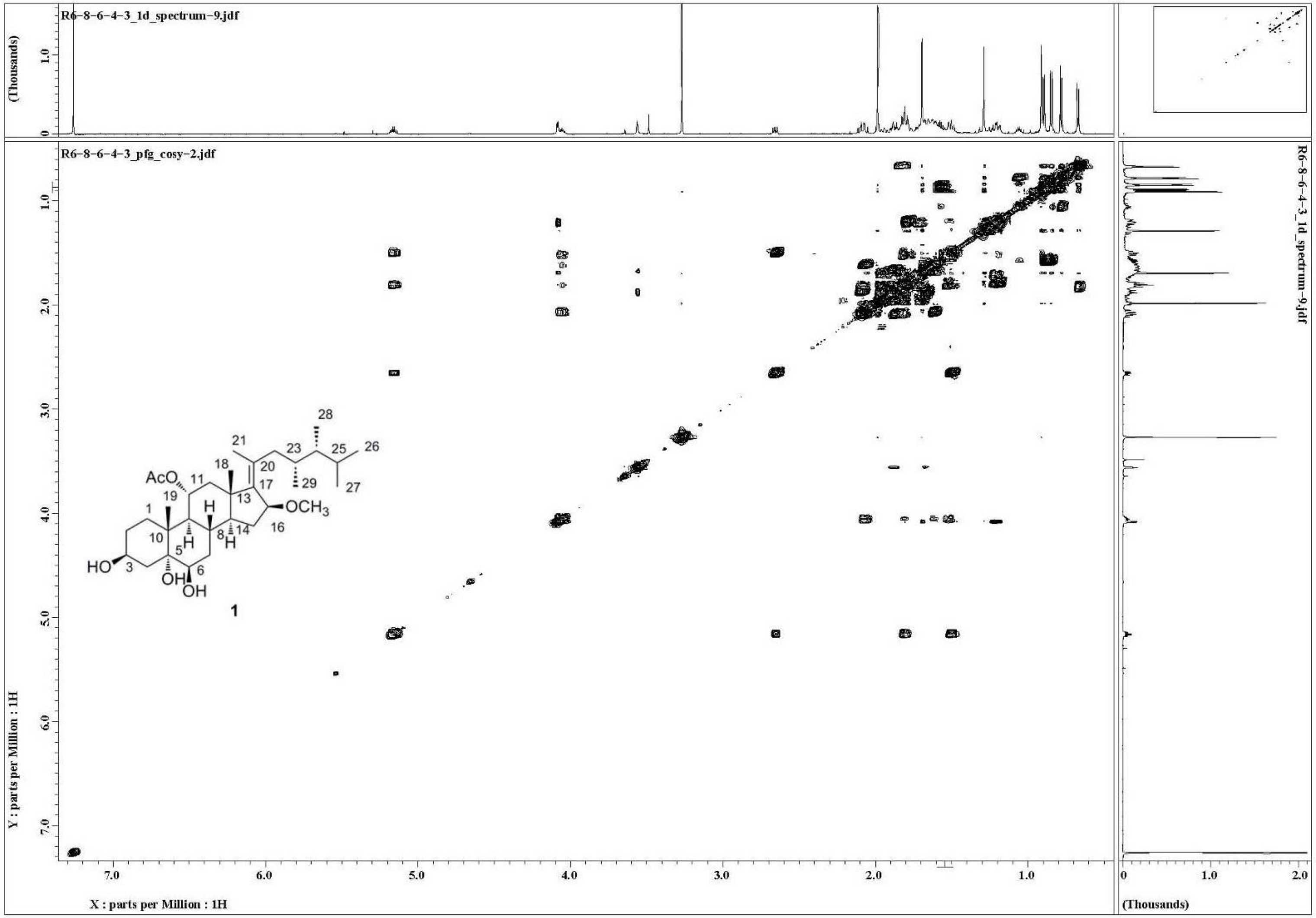

Figure S10. HMQC spectrum of compound (**1**).

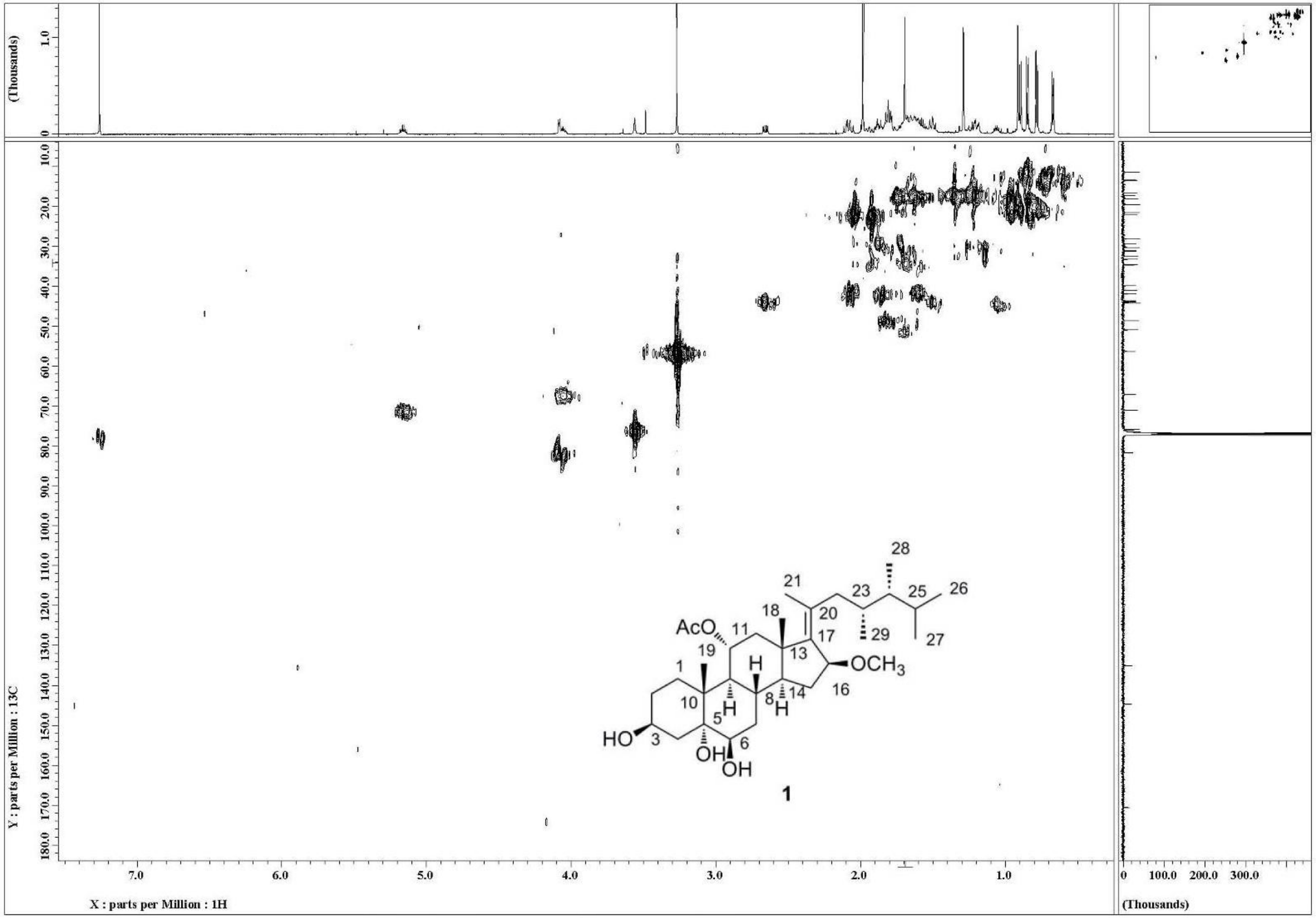

Figure S11. HMBC spectrum of compound (**1**).

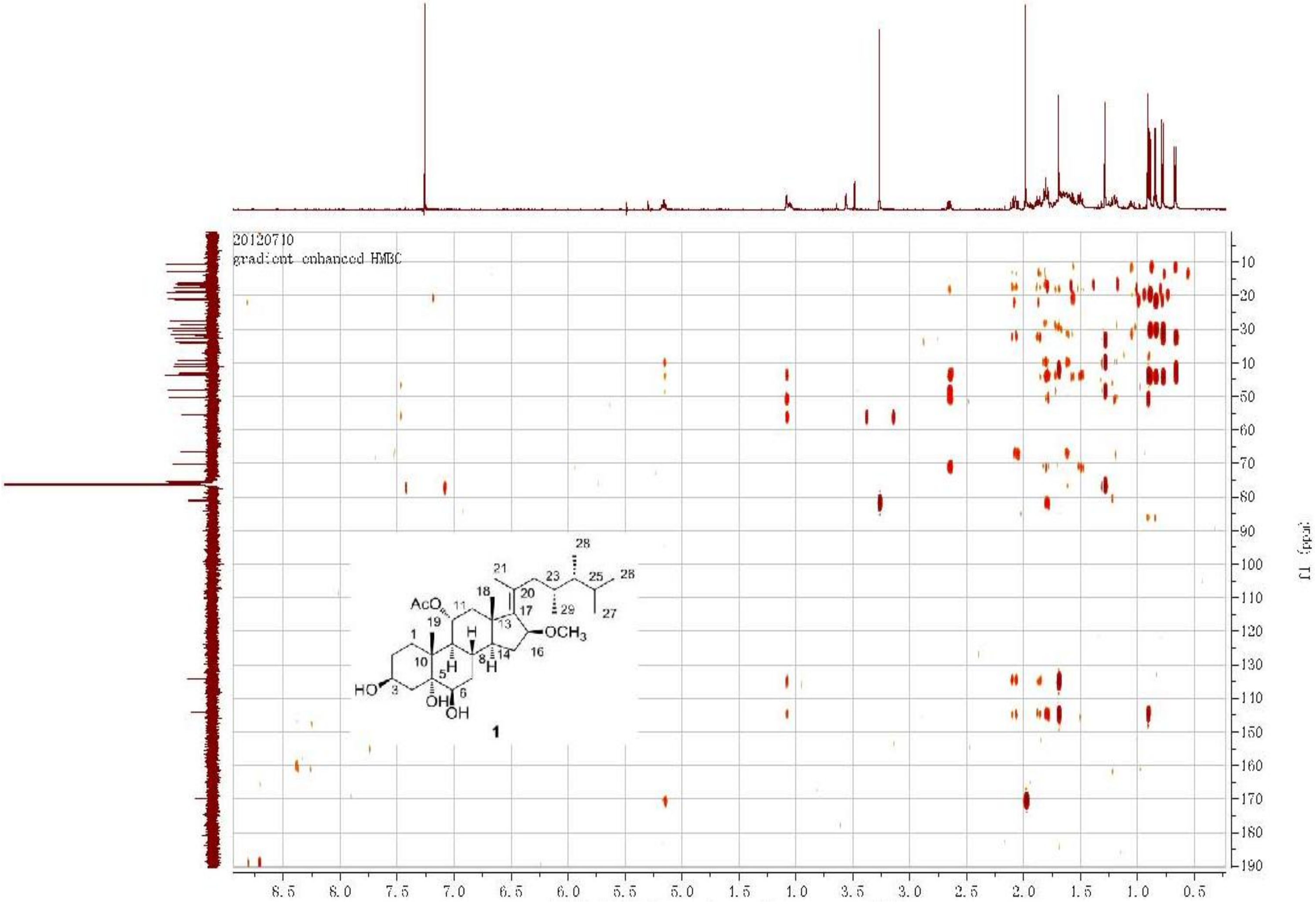

Figure S12. NOESY spectrum of compound (**1**).

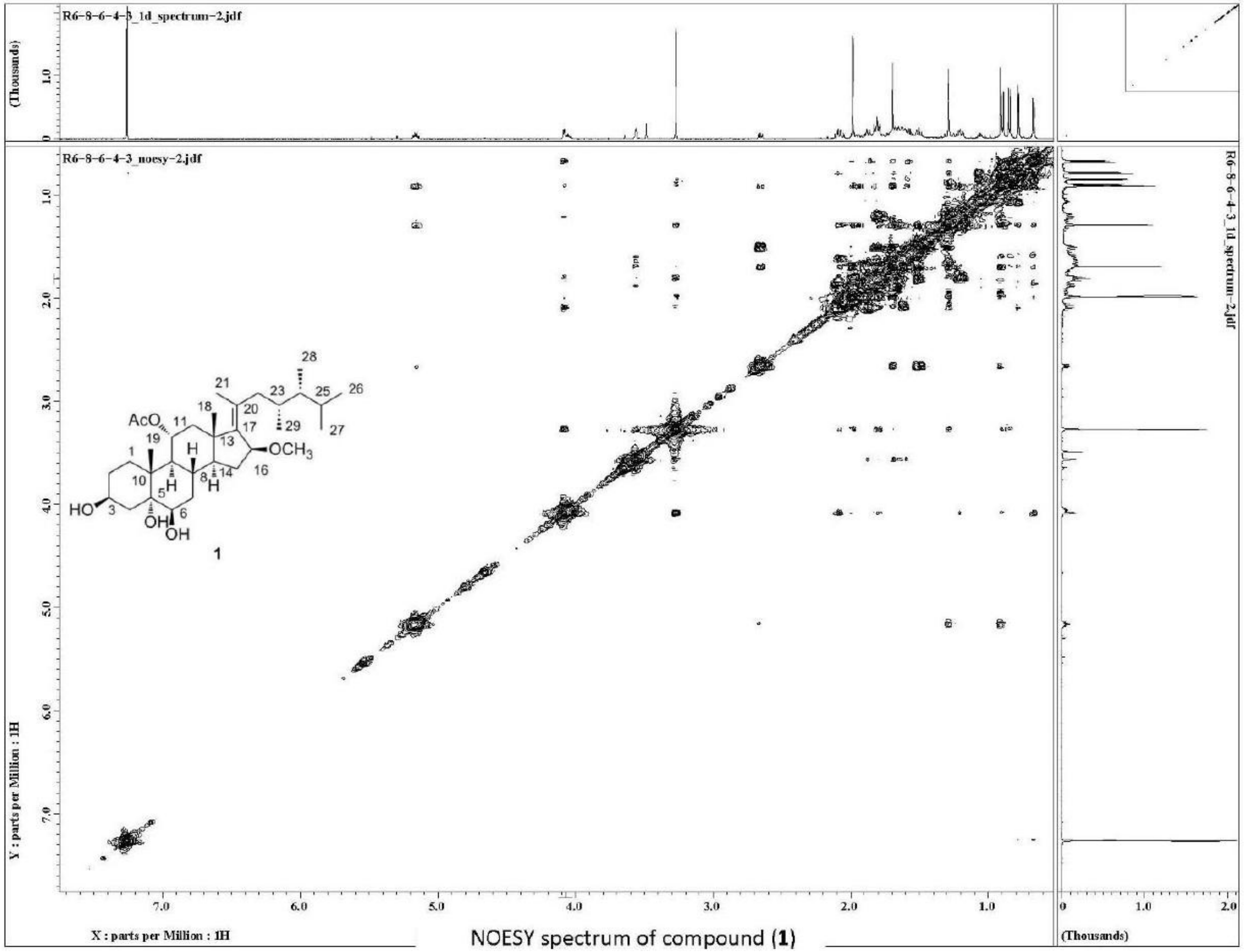

Figure S13. The positive HRESIMS spectrum of compound (**2**).

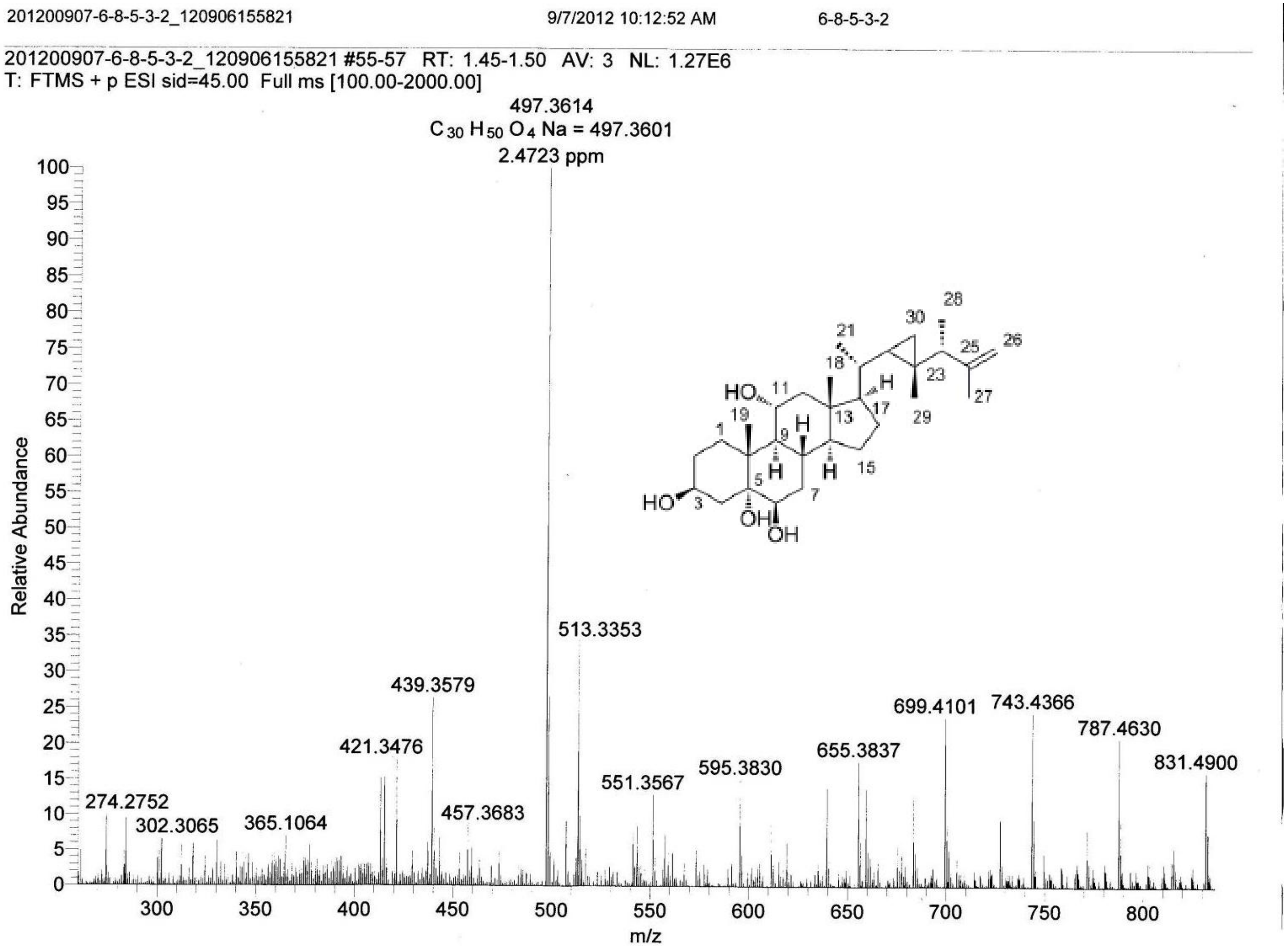

Figure S14. ^{1}H-NMR (600 MHz, C_5D_5N) spectrum of compound (**2**).

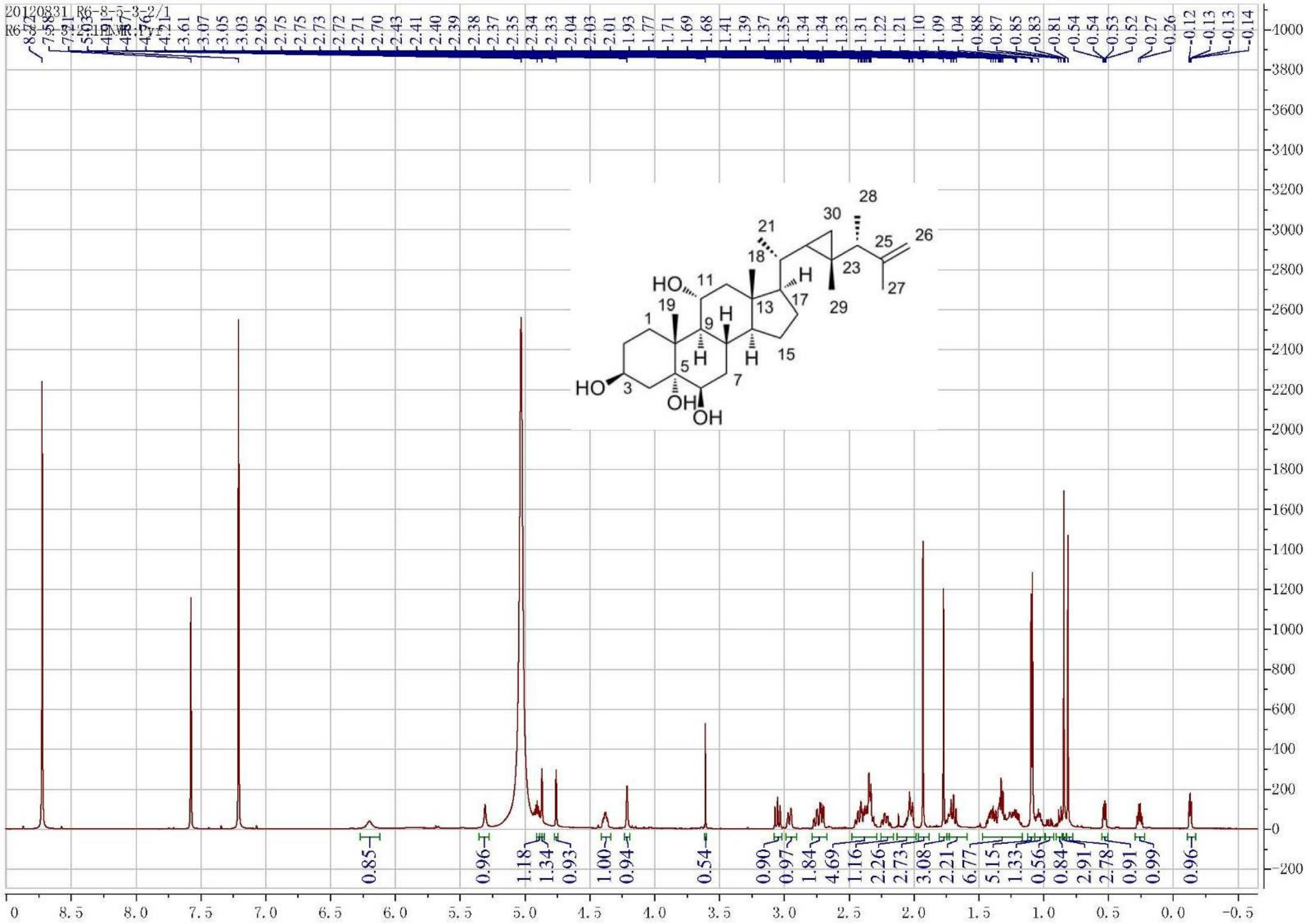

Figure S15. The amplificatory ^{1}H NMR (600 MHz, C_5D_5N) spectrum of compound (**2**).

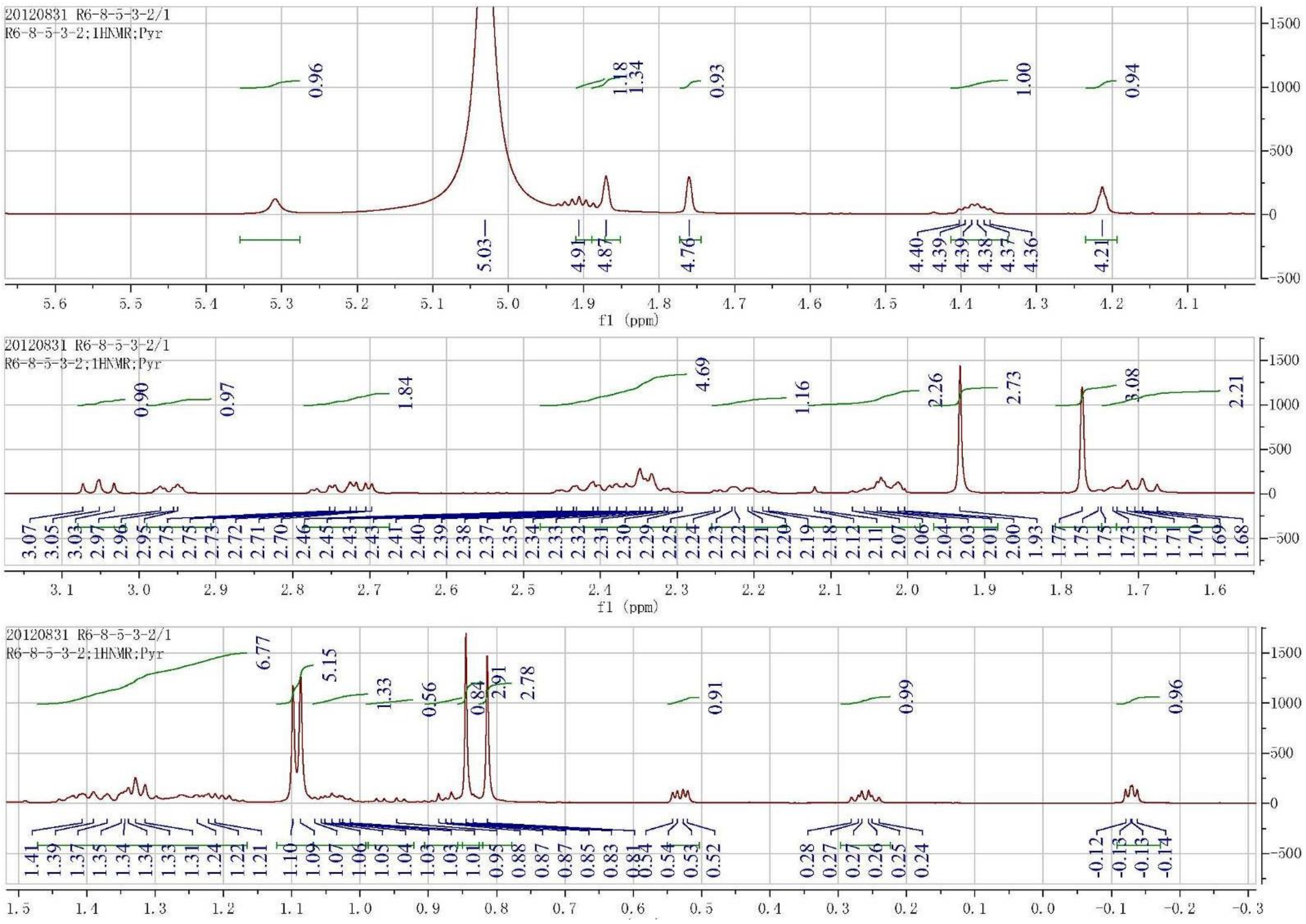

Figure S16. ^{13}C-NMR (150 MHz, C_5D_5N) spectrum of compound (**2**).

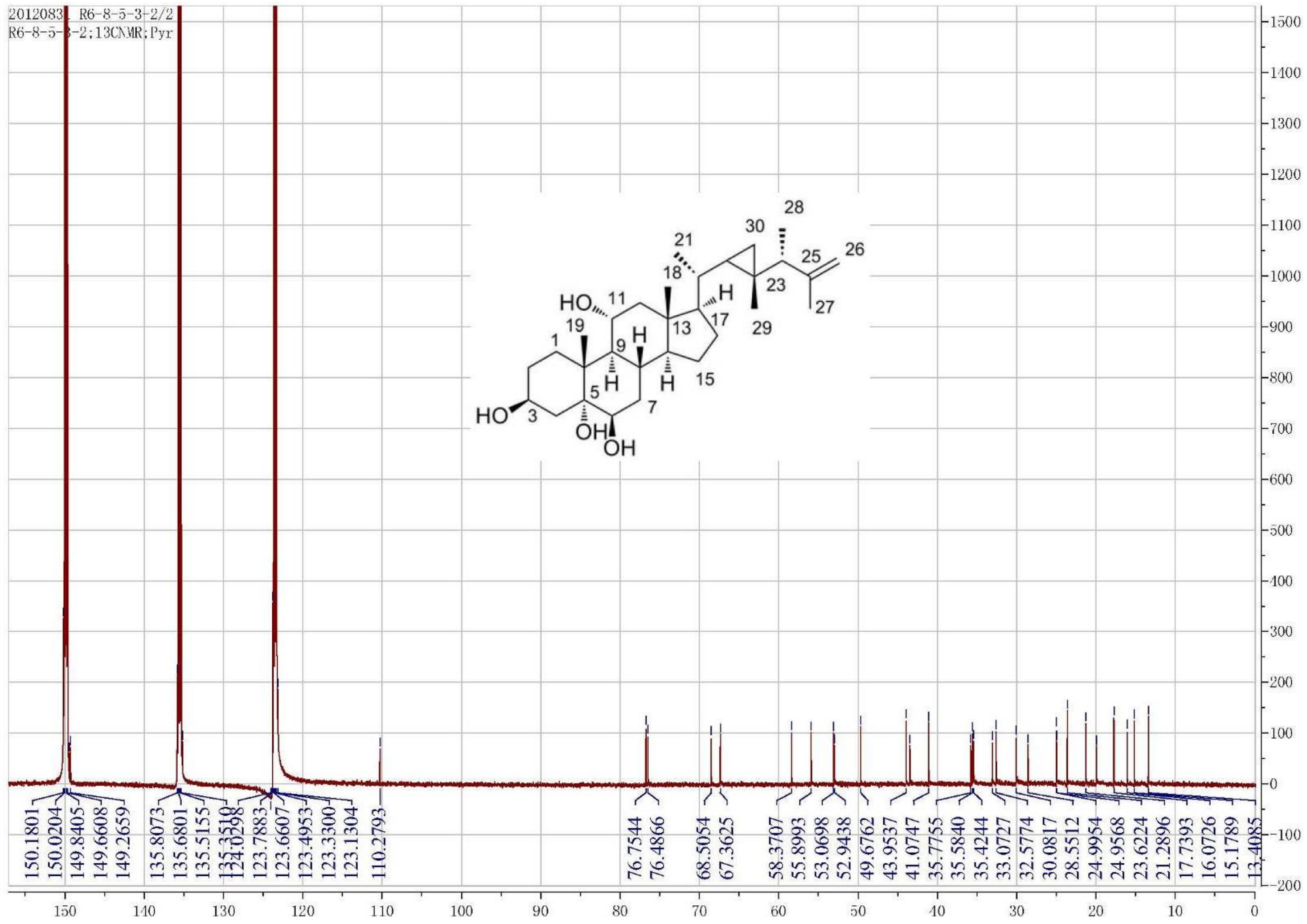

Figure S17. DEPT (150 MHz, C_5D_5N) spectrum of compound (**2**).

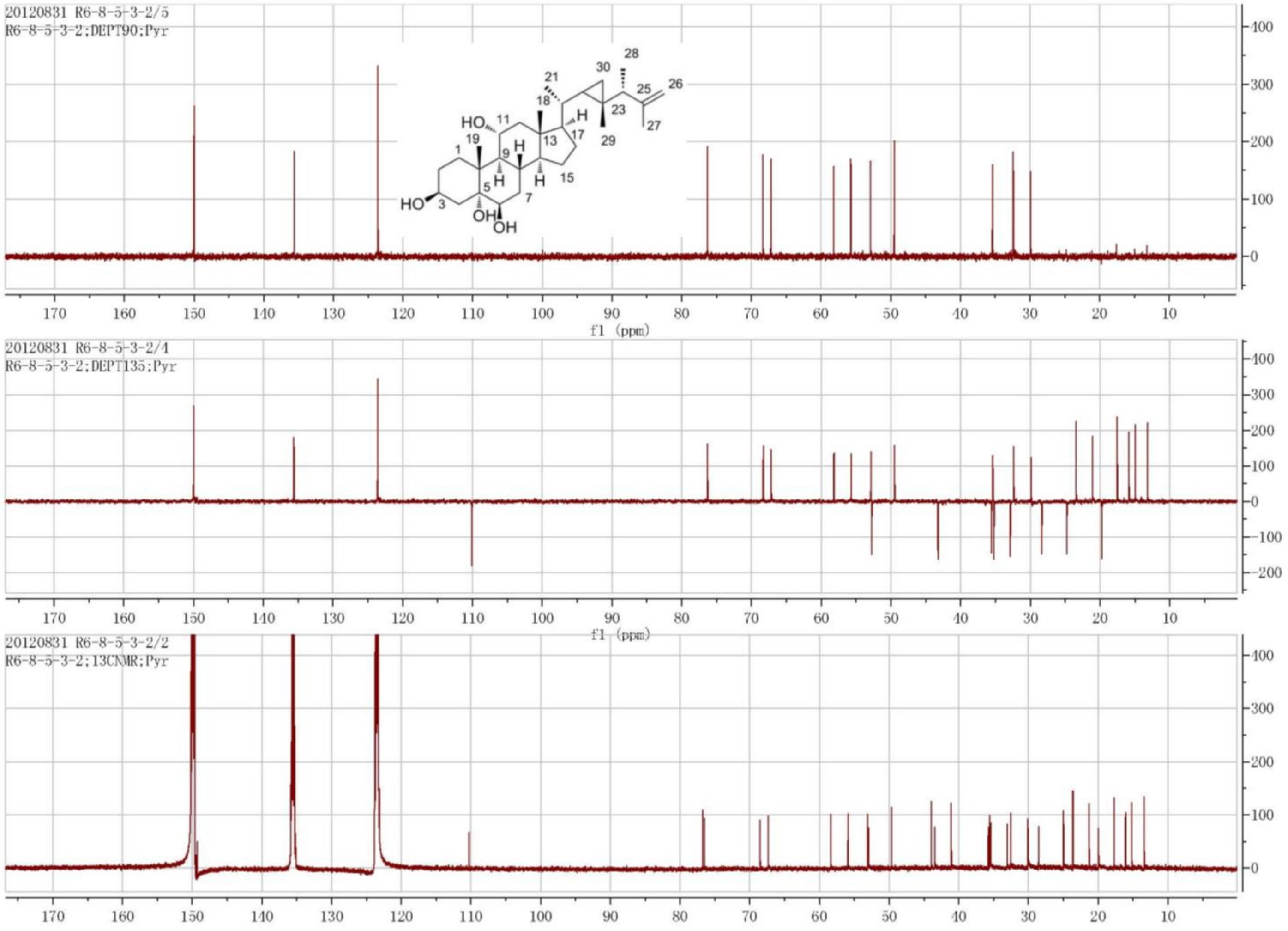

Figure S18. ^{1}H-^{1}H COSY spectrum of compound (**2**).

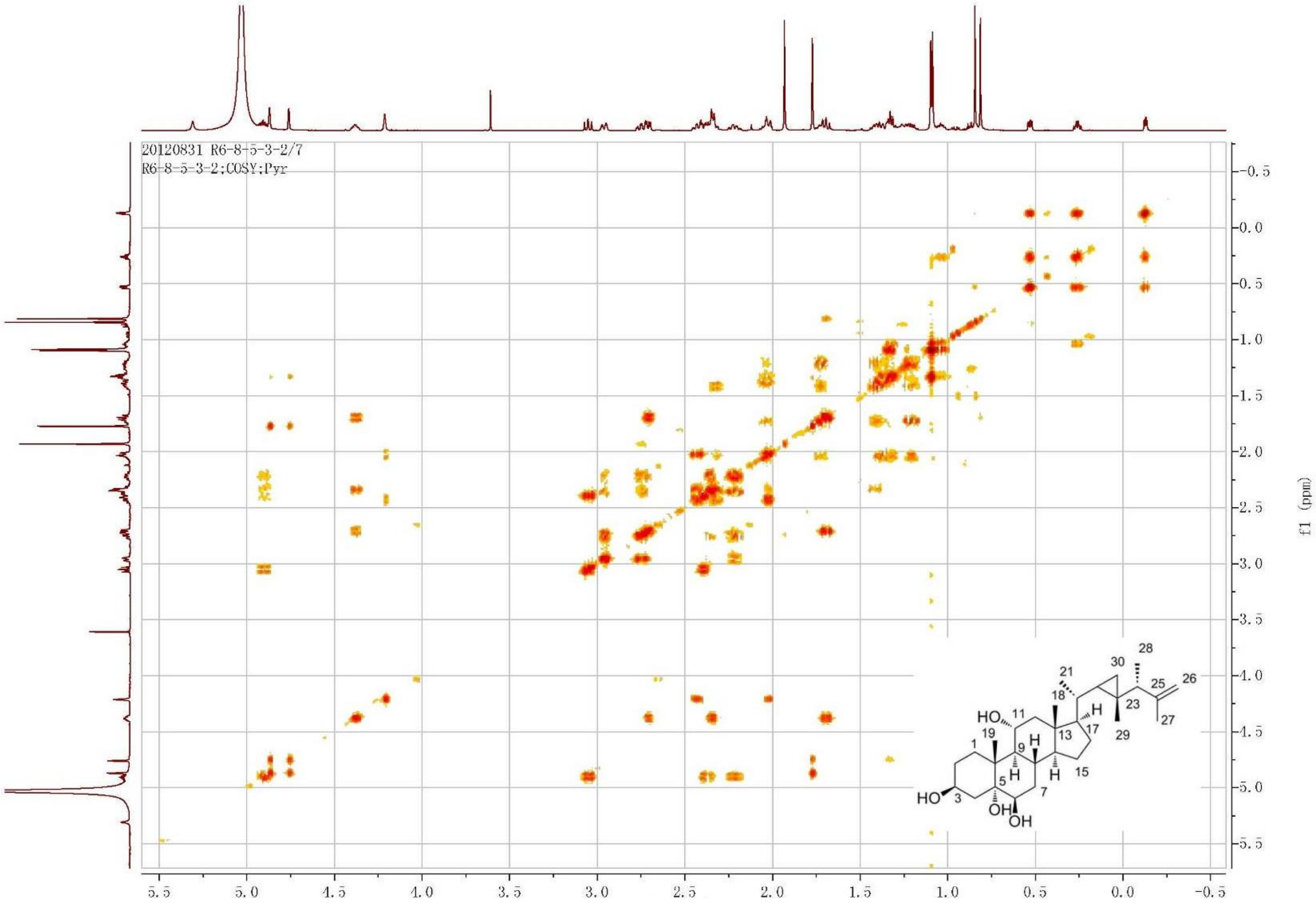

Figure S19. HMQC spectrum of compound (**2**).

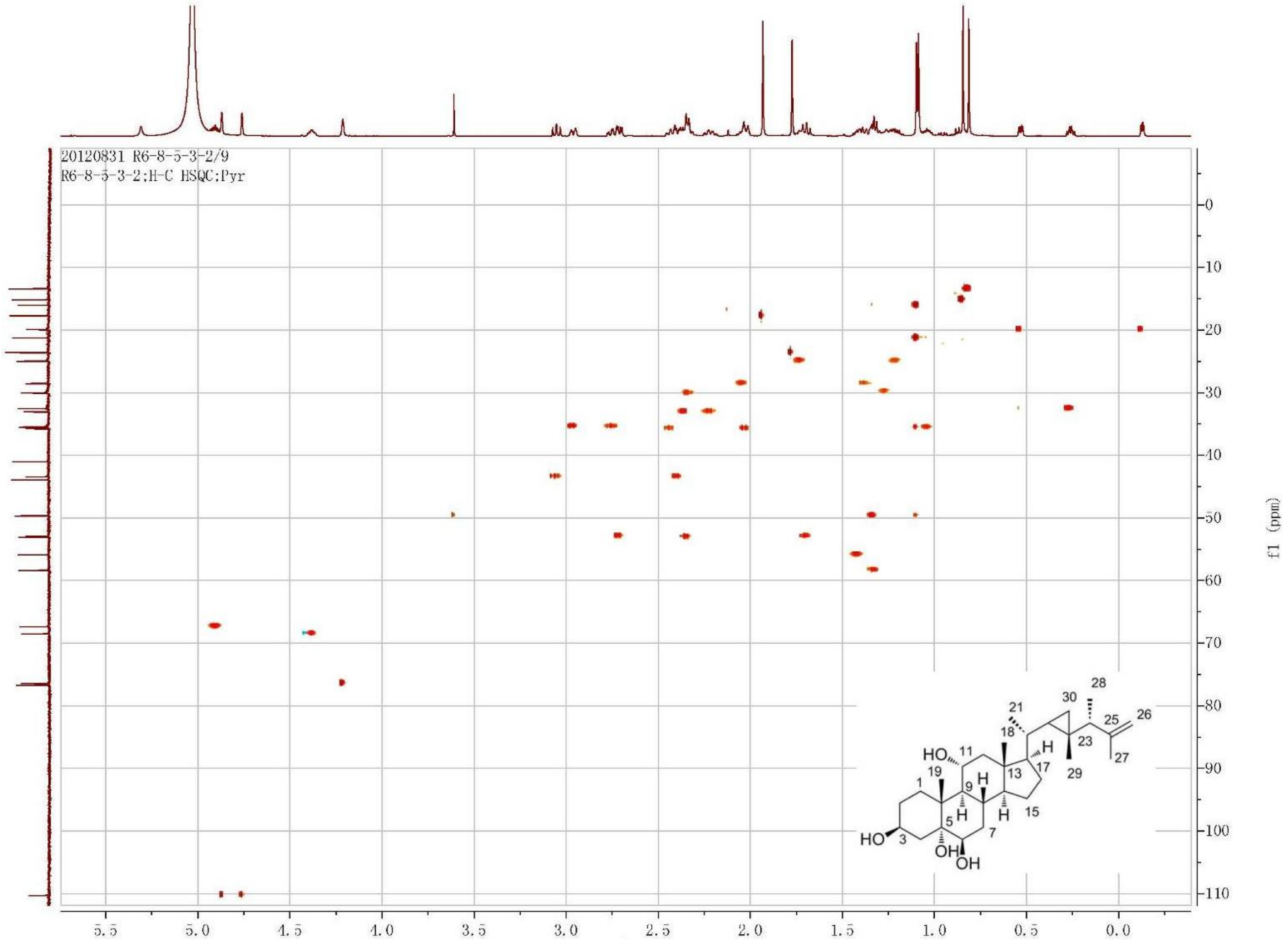

Figure S20. HMBC spectrum of compound (**2**).

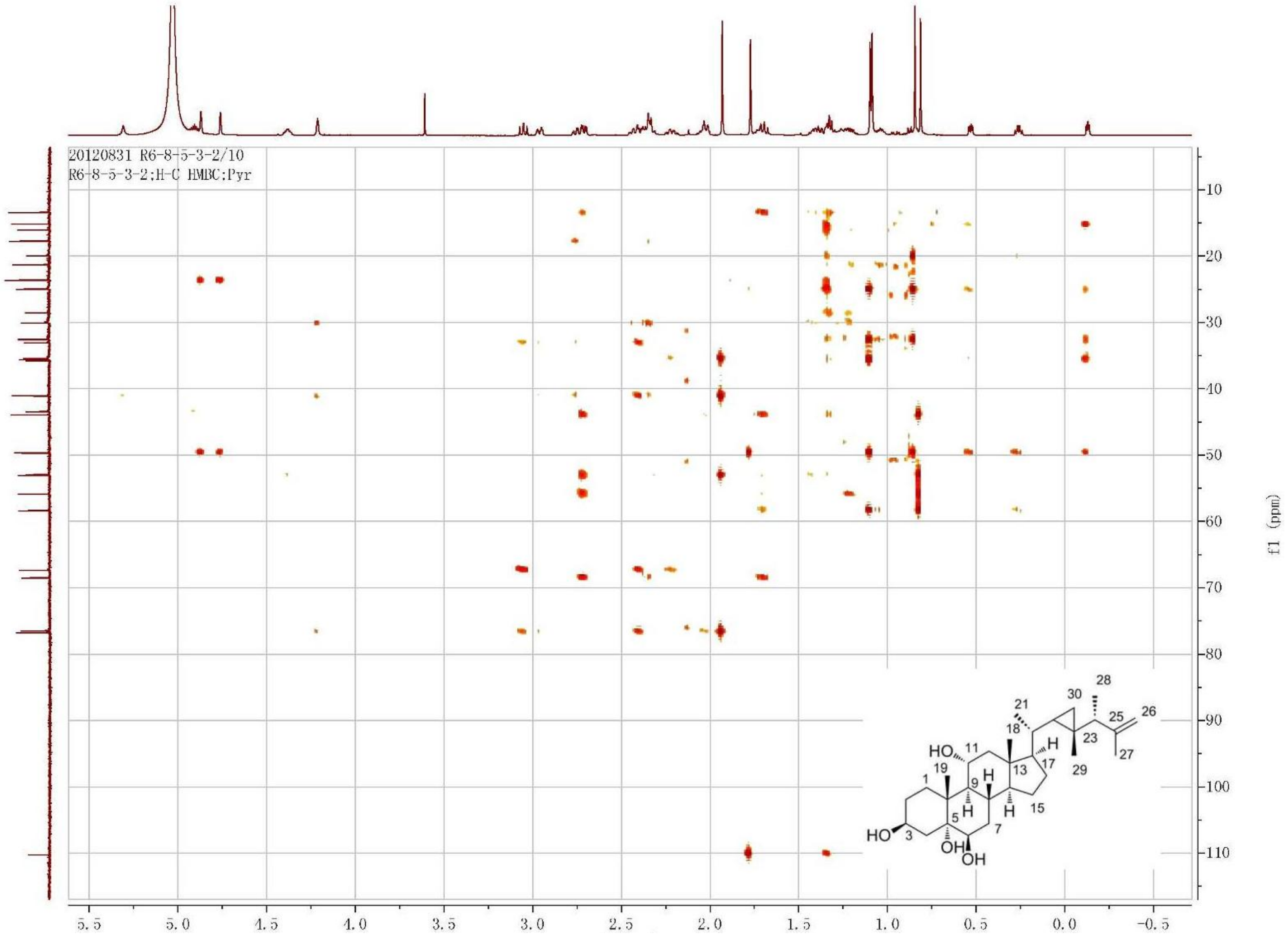

Figure S21 NOESY spectrum of compound (**2**).

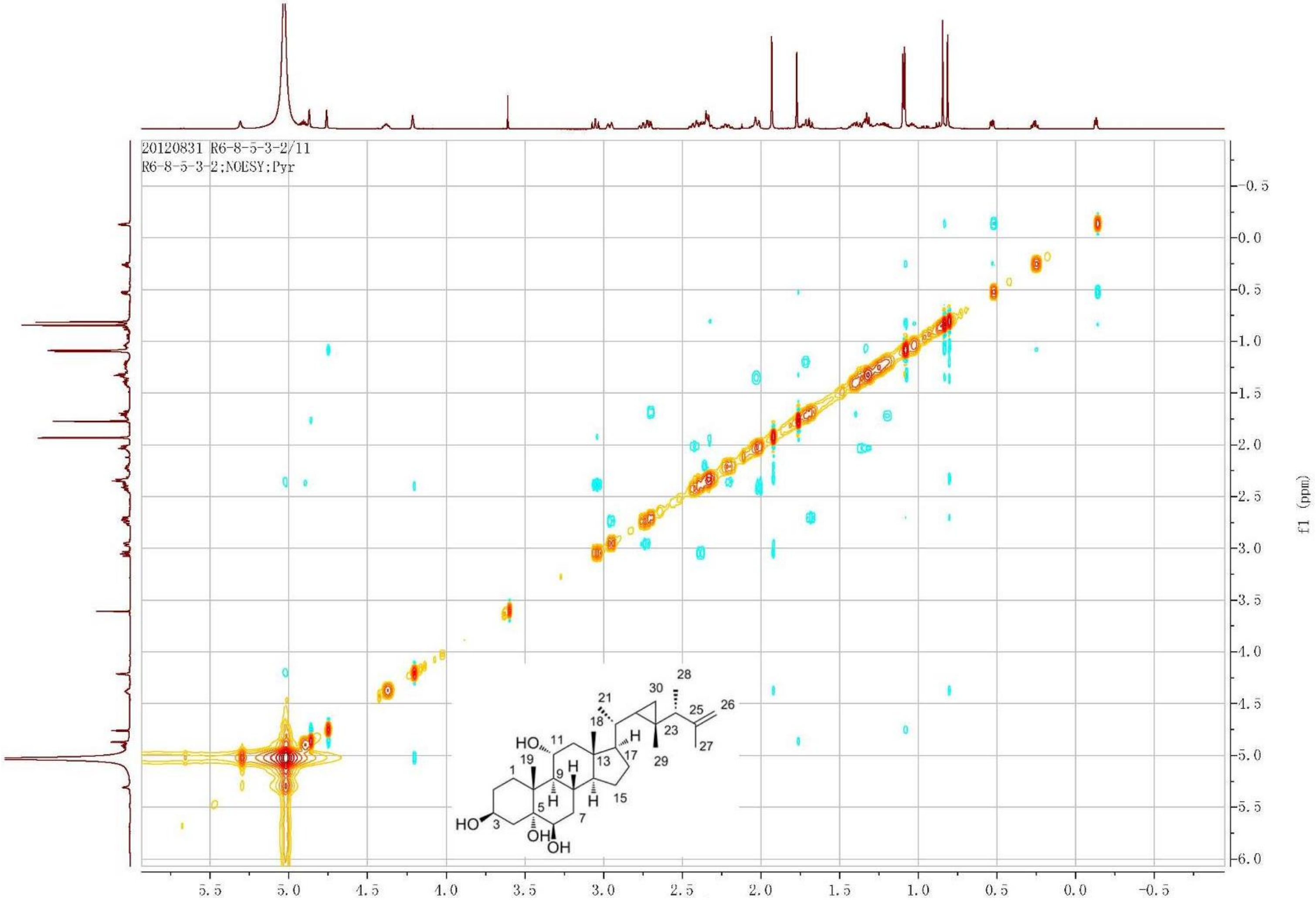

Figure S22. The positive HRESIMS spectrum of compound (**3**).

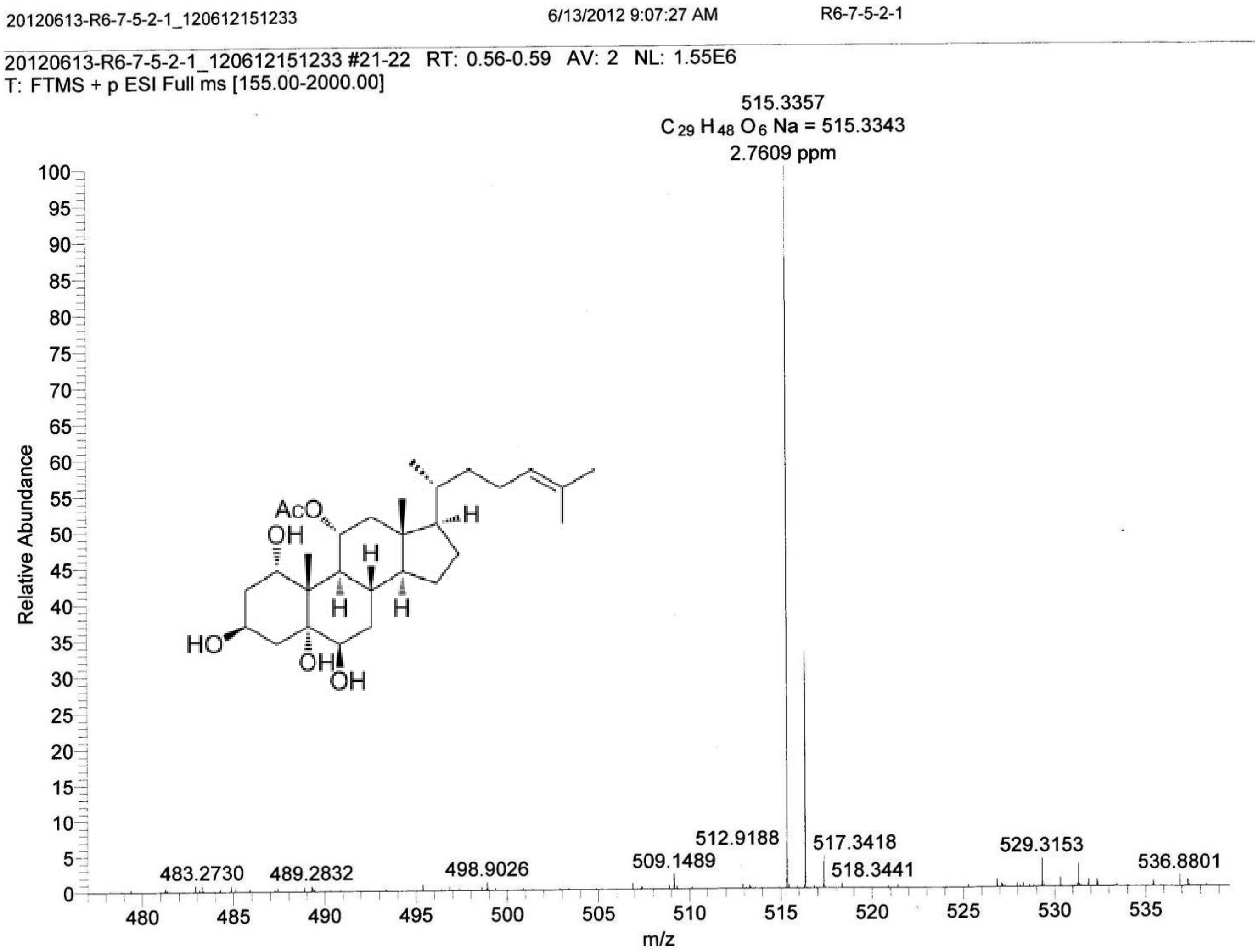

Figure S23. ^{1}H-NMR (600 MHz, $CDCl_3$) spectrum of compound (**3**).

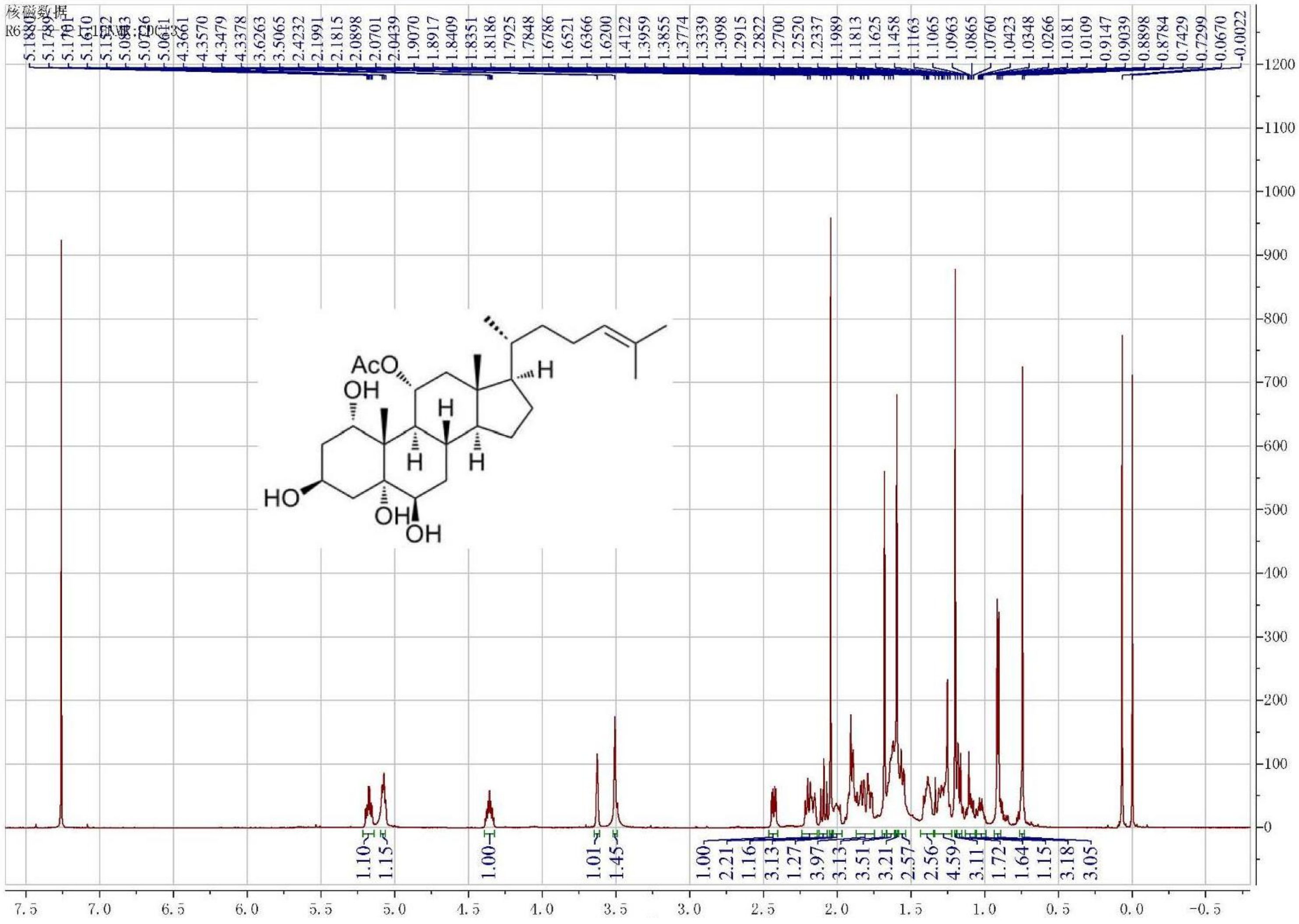

Figure S24. The amplificatory ^{1}H NMR (600 MHz, $CDCl_3$) spectrum of compound (**3**).

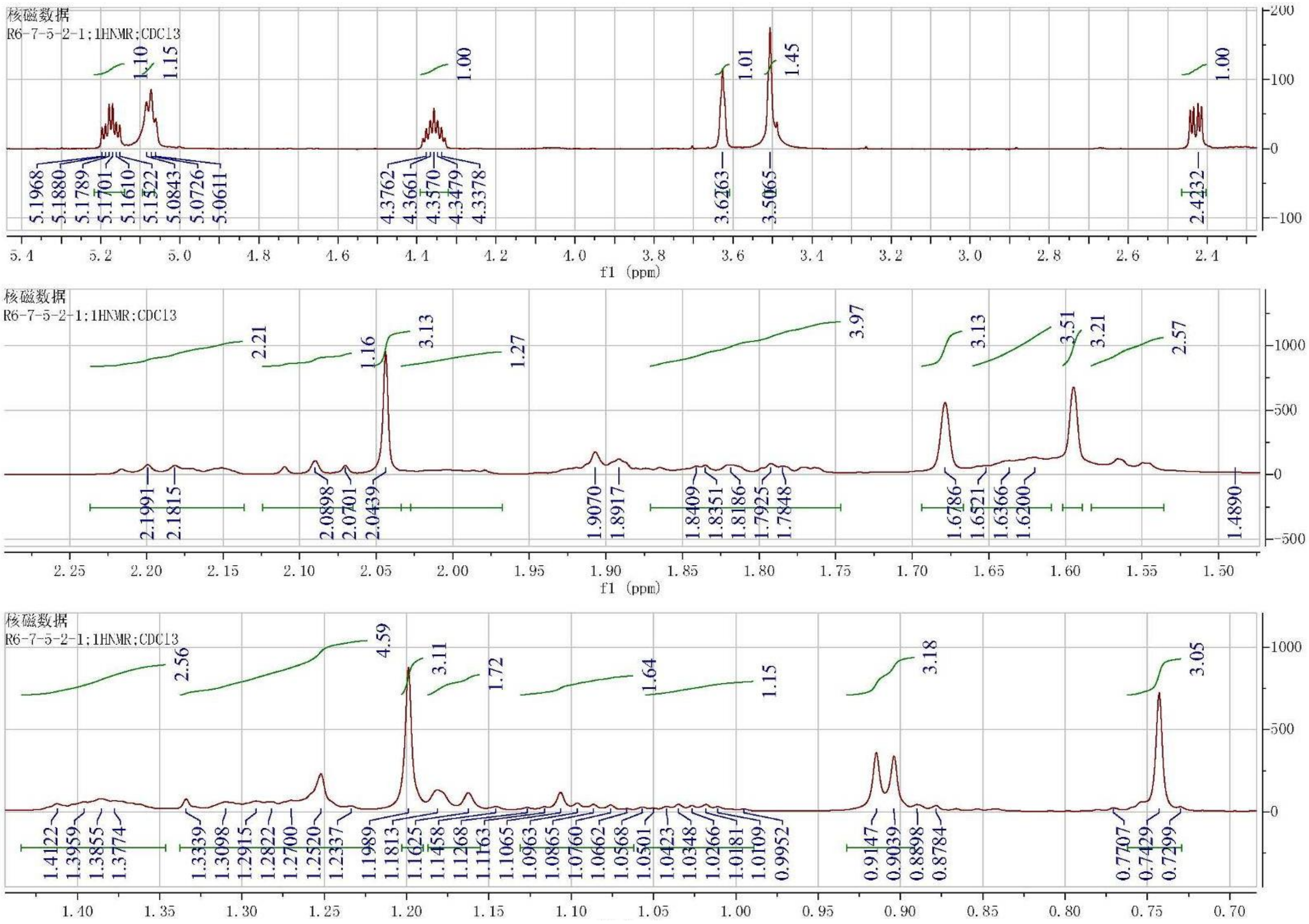

Figure S25. ^{13}C-NMR (150 MHz, $CDCl_3$) spectrum of compound (**3**).

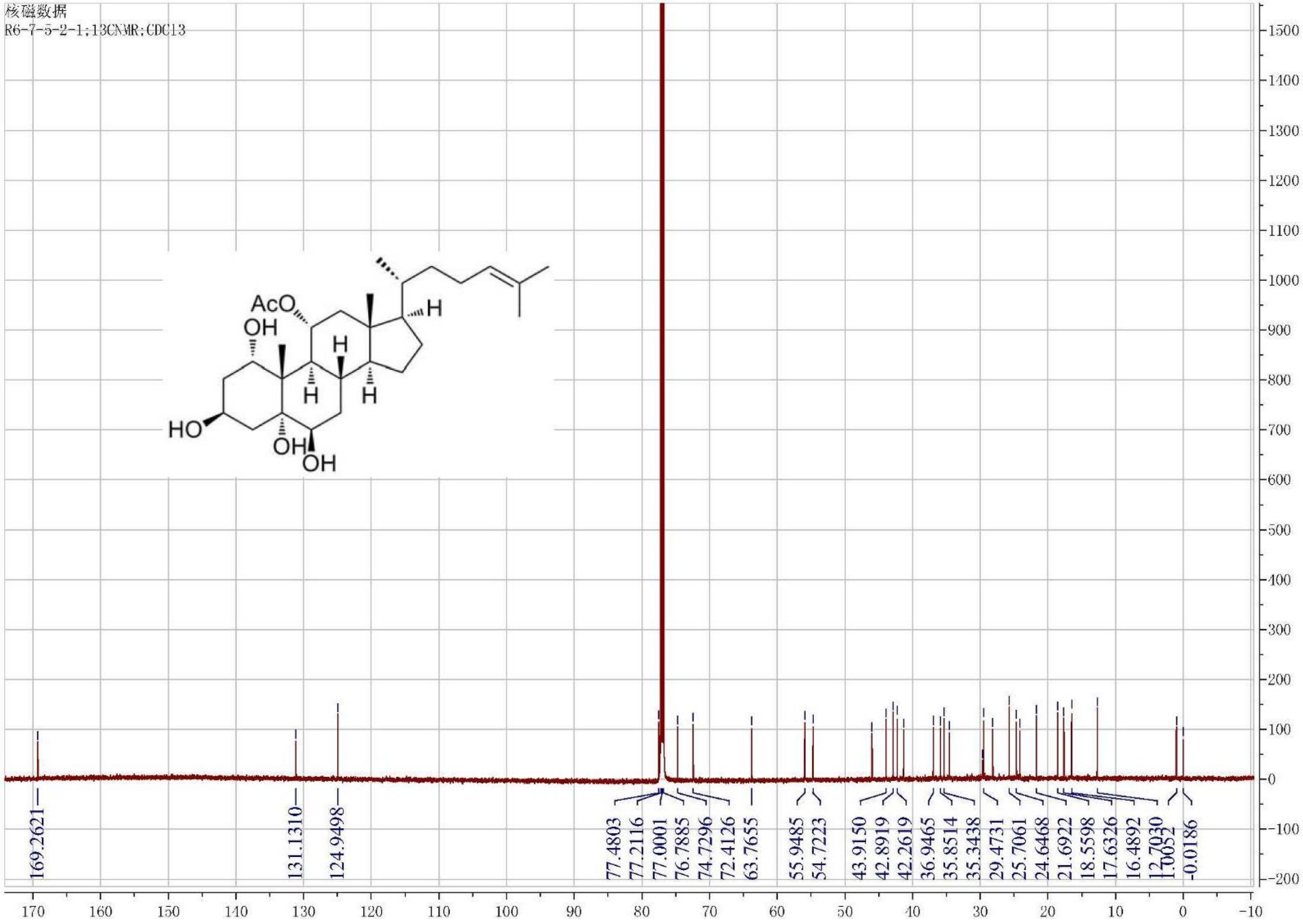

Figure S26. DEPT (150 MHz, $CDCl_3$) spectrum of compound (**3**).

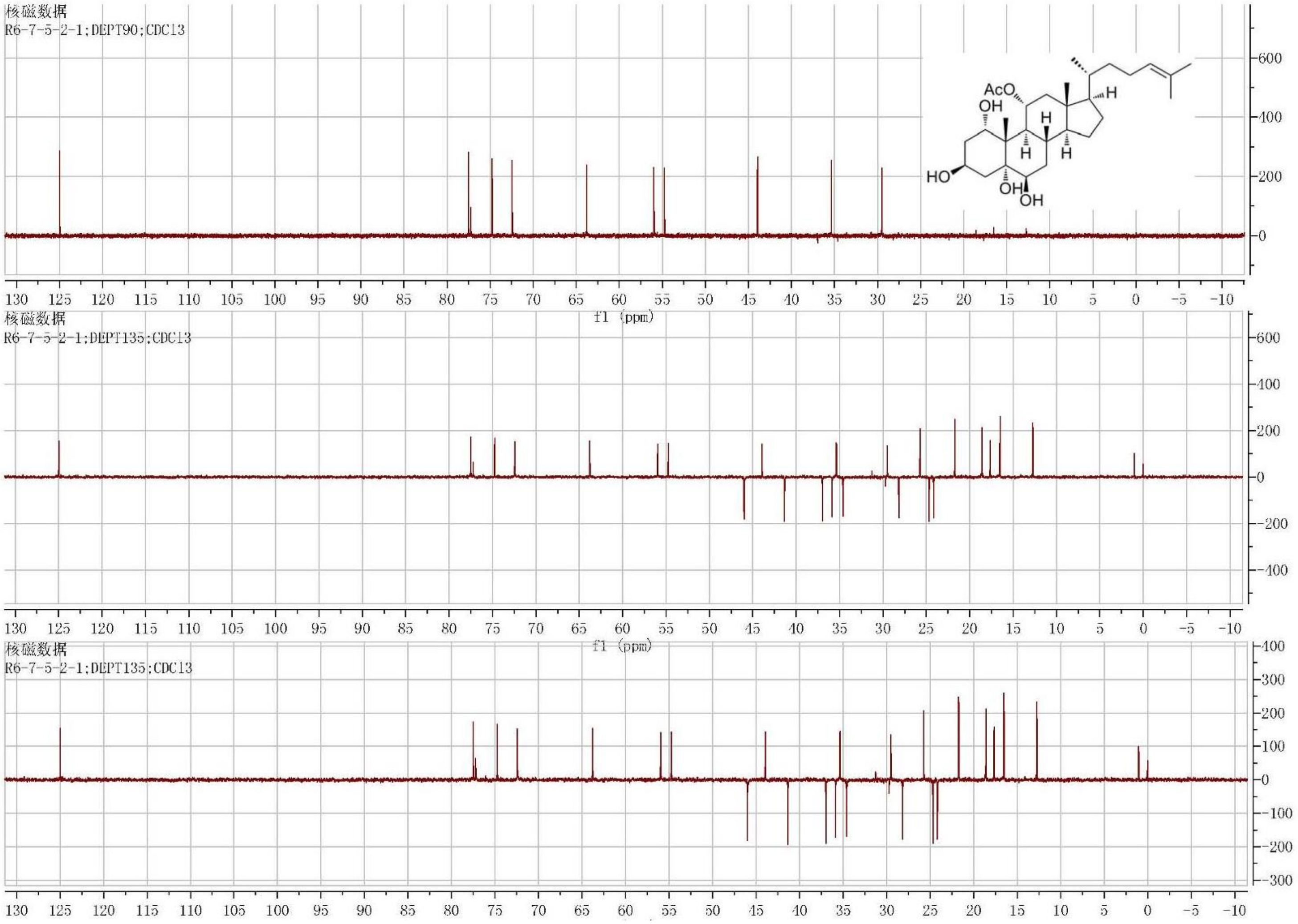

Figure S27. ^{1}H -^{1}H COSY spectrum of compound (**3**).

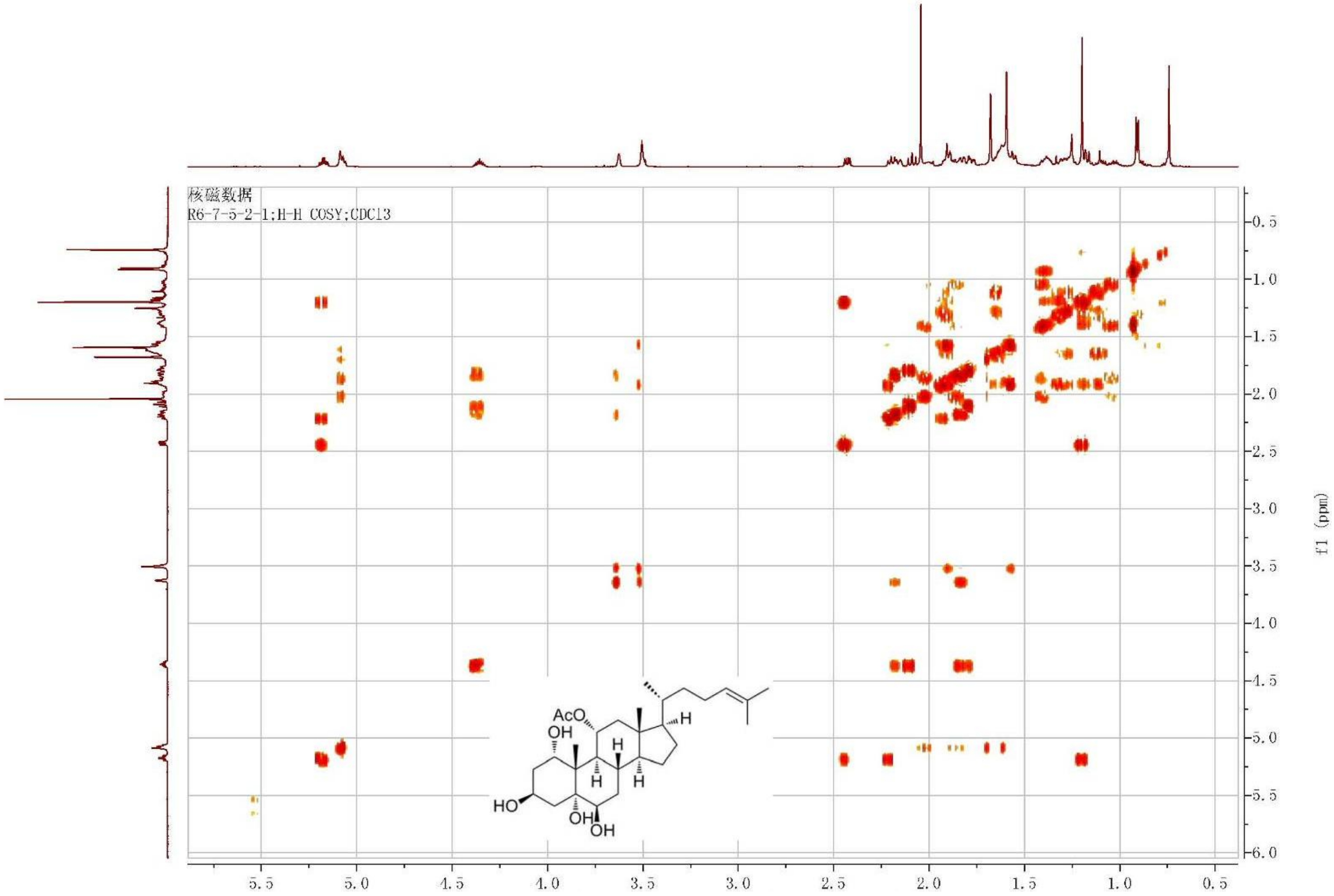

Figure S28. HMQC spectrum of compound (**3**).

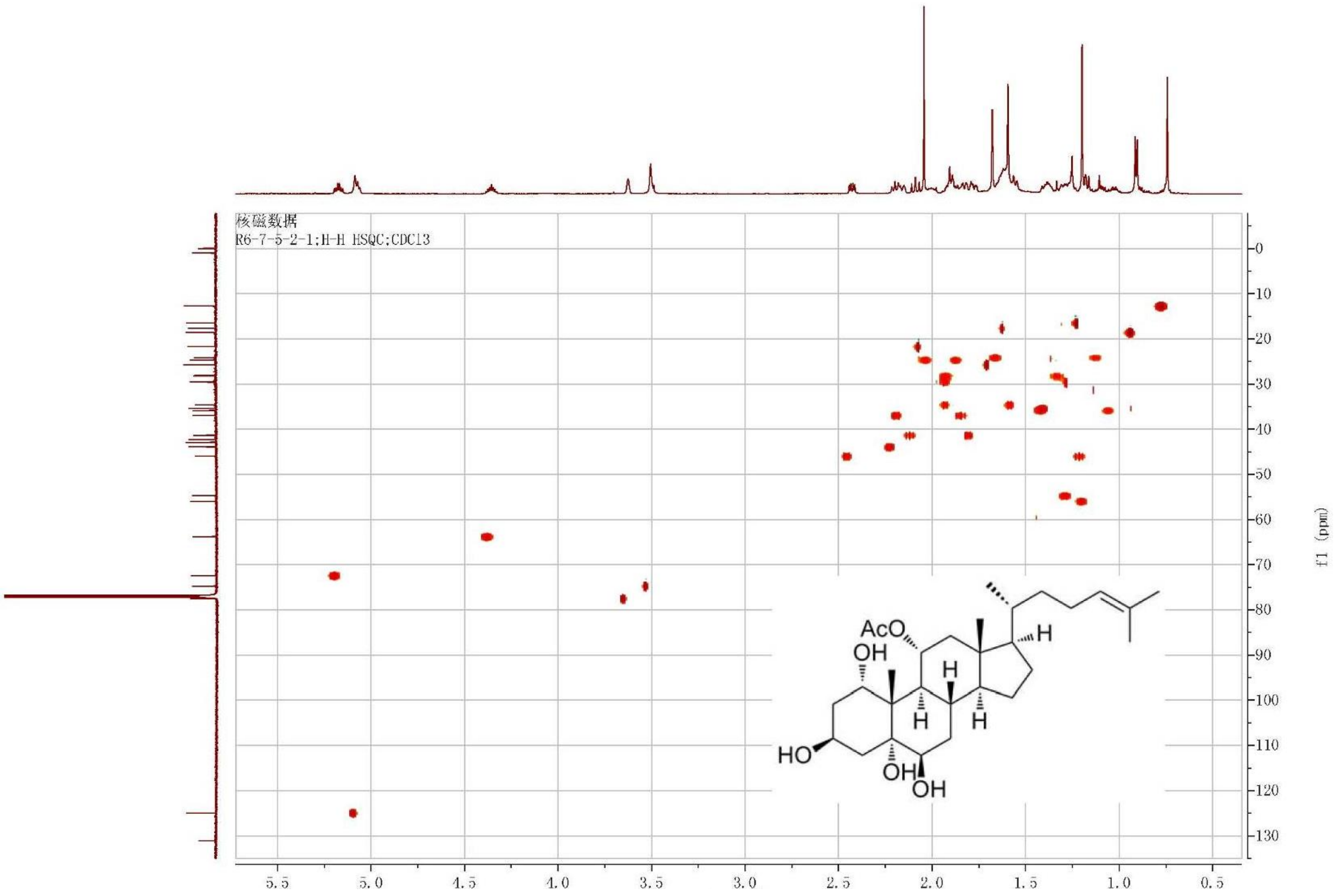

Figure S29. HMBC spectrum of compound (**3**).

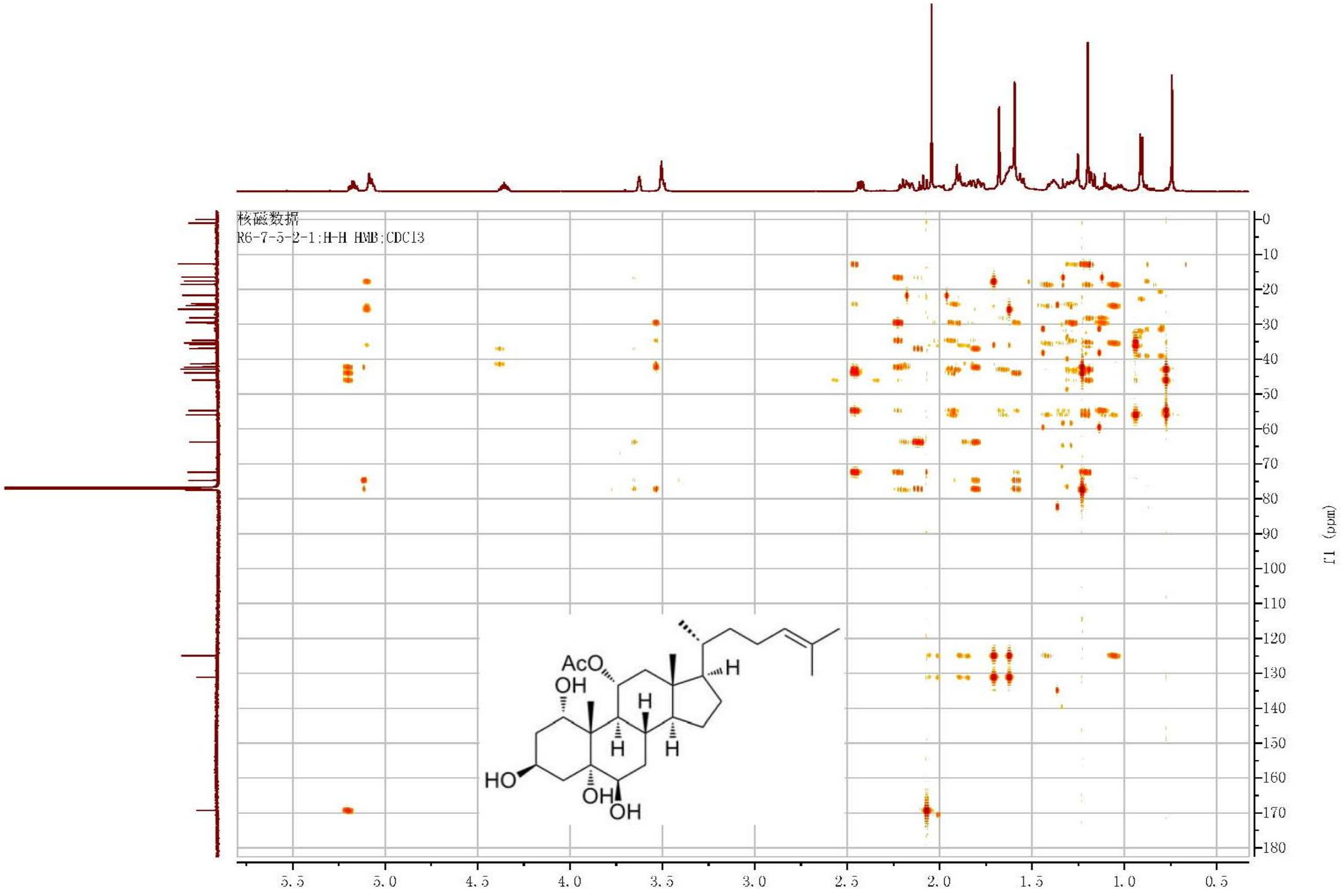

Figure S30. NOESY spectrum of compound (**3**).

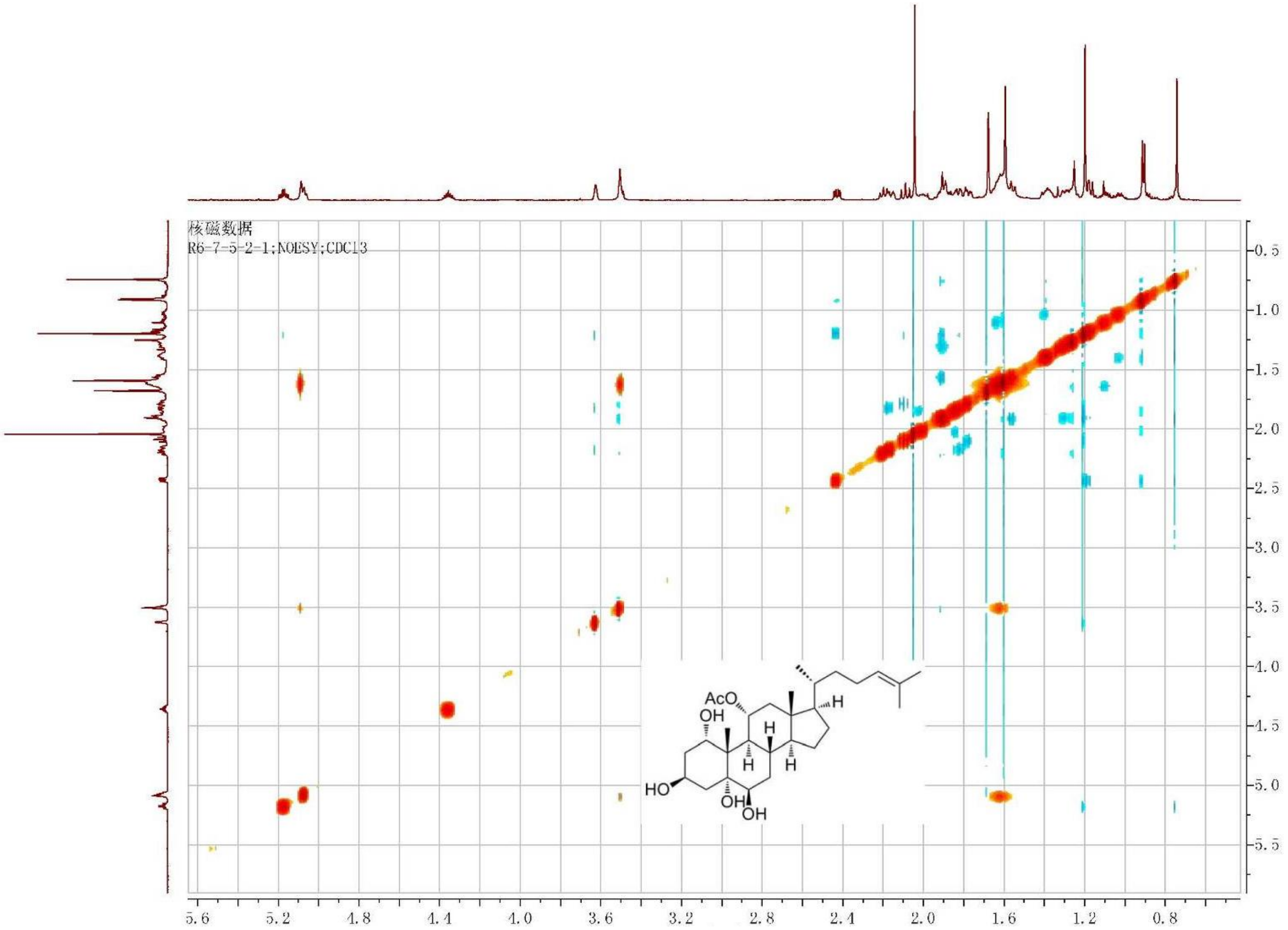

Supplement: Supplementary File 1 — Supplementary Information (PDF, 4789 KB) [file marinedrugs-11-04788-s001.pdf]
